# Production of High Amounts of Hepatotoxin Nodularin and New Protease Inhibitors Pseudospumigins by the Brazilian Benthic *Nostoc* sp. CENA543

**DOI:** 10.3389/fmicb.2017.01963

**Published:** 2017-10-09

**Authors:** Jouni Jokela, Lassi M. P. Heinilä, Tânia K. Shishido, Matti Wahlsten, David P. Fewer, Marli F. Fiore, Hao Wang, Esa Haapaniemi, Perttu Permi, Kaarina Sivonen

**Affiliations:** ^1^Department of Food and Environmental Sciences, University of Helsinki, Helsinki, Finland; ^2^Center for Nuclear Energy in Agriculture, University of São Paulo, Piracicaba, Brazil; ^3^Department of Chemistry, University of Jyväskylä, Helsinki, Finland; ^4^Department of Biological and Environmental Science, Nanoscience Center, University of Jyväskylä, Helsinki, Finland

**Keywords:** cyanobacteria, *Nostoc*, nodularin, spumigin, biosynthesis

## Abstract

*Nostoc* is a cyanobacterial genus, common in soils and a prolific producer of natural products. This research project aimed to explore and characterize Brazilian cyanobacteria for new bioactive compounds. Here we report the production of hepatotoxins and new protease inhibitors from benthic *Nostoc* sp. CENA543 isolated from a small, shallow, saline-alkaline lake in the Nhecolândia, Pantanal wetland area in Brazil. *Nostoc* sp. CENA543 produces exceptionally high amounts of nodularin-R. This is the first free-living *Nostoc* that produces nodularin at comparable levels as the toxic, bloom-forming, *Nodularia spumigena*. We also characterized pseudospumigins A–F, which are a novel family of linear tetrapeptides. Pseudospumigins are structurally related to linear tetrapeptide spumigins and aeruginosins both present in *N. spumigena* but differ in respect to their diagnostic amino acid, which is Ile/Leu/Val in pseudospumigins, Pro/mPro in spumigins, and Choi in aeruginosins. The pseudospumigin gene cluster is more similar to the spumigin biosynthetic gene cluster than the aeruginosin gene cluster. Pseudospumigin A inhibited trypsin (IC_50_ 4.5 μM after 1 h) in a similar manner as spumigin E from *N. spumigena* but was almost two orders of magnitude less potent. This study identifies another location and environment where the hepatotoxic nodularin has the potential to cause the death of eukaryotic organisms.

## Introduction

**Figure 1:**
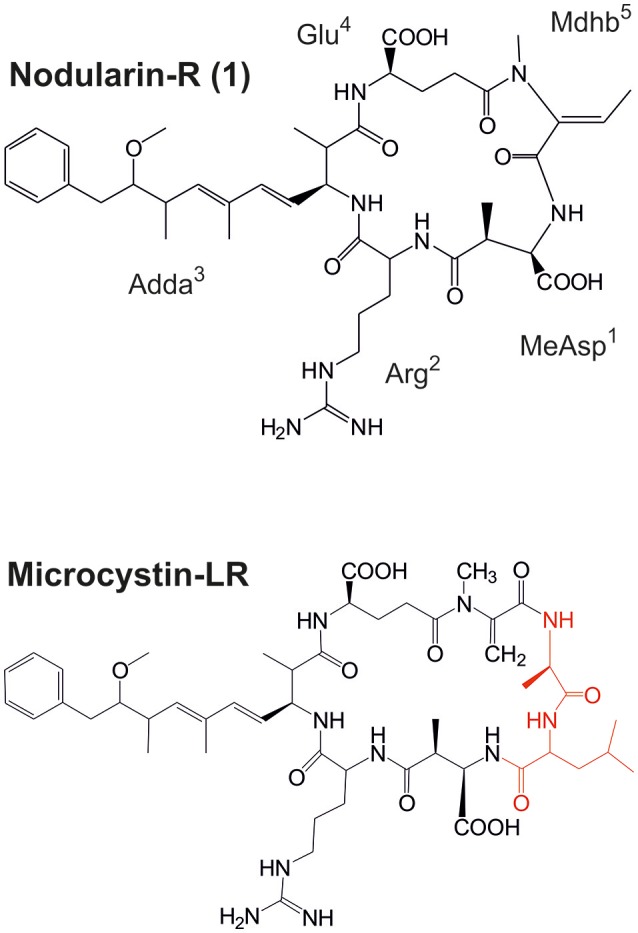
Structures of nodularin-R and microcystin-LR, which is the most common variant of microcystin (amino acids marked red are not present in nodularins). MeAsp, D-*erythro*-β-methylaspartic; Adda, 3-amino-9-methoxy-10-phenyl-2,6,8-trimethyl-deca-4(E),6(E)-dienoic acid; Mdhb, N-methyl-dehydrobutyric acid.

*Nostoc* is a cosmopolitan genus of cyanobacteria that is commonly found in soil and in symbiotic associations with plants and fungi. Members of the genus *Nostoc* are prolific producers of natural products including peptides, polyketides, and alkaloids (Kobayashi and Kajiyama, 1998; Dittmann et al., 2015). Free-living and symbiotic strains of *Nostoc* are known to produce a range of microcystins, potent cyclic heptapeptide hepatotoxins produced by a number of cyanobacteria (Sivonen et al., 1990; Oksanen et al., 2004; Genuário et al., 2010; Kaasalainen et al., 2012; Figure 1). Nodularin (NOD) is a hepatotoxic protein phosphatase inhibitor and tumor promoter that has two amino acid residues fewer than microcystins (Figure 1). More than 240 structural variants have been reported for microcystins but only 10 structural variants of NOD are known to date (Meriluoto et al., 2017). This disparity can be partly explained by the most variable amino acid in microcystins being absent from NODs. Sivonen et al. (1990) had reported that *Nostoc* is one of the cyanobacterial genera that produce microcystins and since then microcystin production have been reported to occur in several other *Nostoc* strains (Oksanen et al., 2004; Genuário et al., 2010; Kaasalainen et al., 2012). NOD was detected only in free living *Nodularia* until 2012 when NOD was also found in cycad endosymbiotic *Nostoc* strains in low concentrations (<0.4 μg/g biomass) and also in liverwort symbiotic *Nostoc* sp. SKS8 (Gehringer et al., 2012; Liaimer et al., 2016). Higher amounts of NOD have been found among the cyanolichens, in which *Nostoc* is the most common cyanobacterial endosymbiont (Kaasalainen et al., 2012).

The cyanobacterium *Nodularia spumigena* that forms blooms in brackish water can produce NOD at high amounts, >5 mg NOD/g biomass dry weight (DW; Repka et al., 2001). These blooms constitute a health risk for human and domestic animals (Sivonen, 2009; Simola et al., 2012). The latest report of a new NOD producer is from tropical Australia, where a new cyanobacterial species *Iningainema pulvinus* gen nov., sp. nov. was isolated from a freshwater wetland spring (McGregor and Sendall, 2017). *I. pulvinus* is the third genus from which NOD production has been reported. The mean level was 0.9 mg NOD g^−1^ biomass DW, which is over three orders of magnitude higher than the amount of NOD previously reported from *Nostoc* (Gehringer et al., 2012).

**Figure 2:**
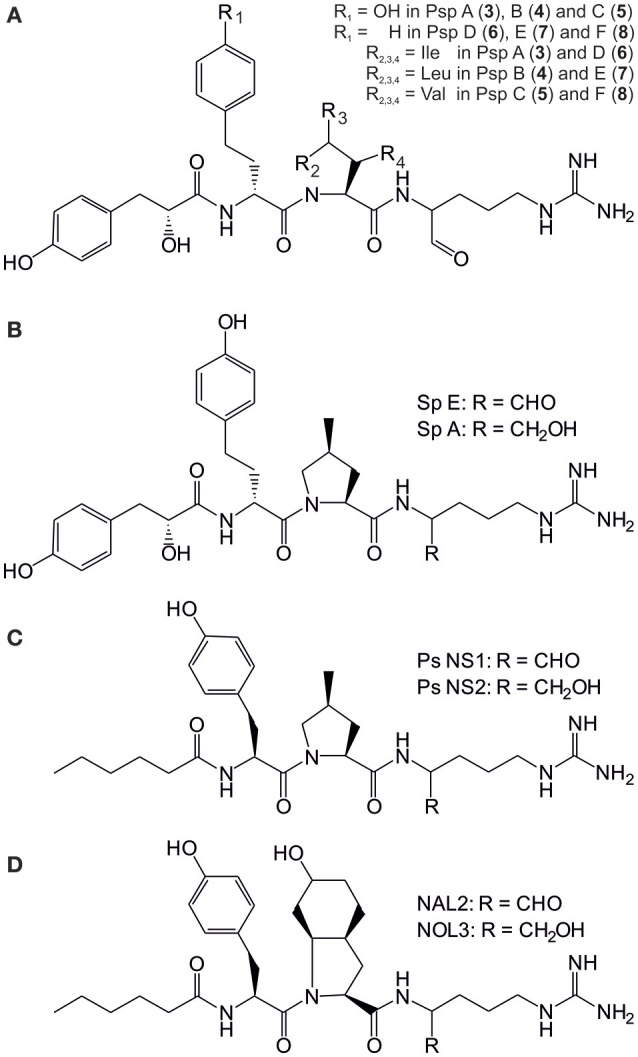
Structures of the pseudospumigins (Psp) A–E (**3**–**8**) produced by *Nostoc* sp. CENA543 **(A)** and *Nodularia spumigena* produced spumigins (Sp) E and A **(B)**, pseudoaeruginosins (Ps) NS1 and NS2 **(C)** and aeruginosins NAL2 and NOL3 **(D)**.

Spumigins and aeruginosins are linear peptide protease inhibitors that comprise three amino acids and a terminal carboxylic acid residue (Fewer et al., 2009, 2013). The presence or absence of the diagnostic amino acids, Pro/mPro in spumigins and the amino acid Choi (2-carboxy-6-hydroxyoctahydroindole) in aeruginosins, are the chemical determining entities that differentiates these two peptide groups (Figure 2). These peptide groups are products of non-ribosomal peptide synthetase biosynthetic pathways that do share a great deal of structural similarities (Fewer et al., 2009, 2013). Spumigins were first described in *N. spumigena* AV1 (Fujii et al., 1997). Twenty-four spumigin congeners in total have been described. Spumigins A-I and nine other spumigins have been described from *N. spumigena* strains isolated in the Baltic Sea and also in Turkish freshwater lake (Fujii et al., 1997; Fewer et al., 2009; Mazur-Marzec et al., 2013). Moreover, spumigin J from *Anabaena compacta* isolated from Lake Esthwaite Water in England (Anas et al., 2012) and four spumigin congeners from *Sphaerospermopsis torques-reginae* cyanobacterium isolated from a bloom sample collected in the Tapacurá reservoir in Brazil have been described (Sanz et al., 2015). Aeruginosins are more common and roughly 100 variants that show a higher structural variation compared to spumigins have been described (Dittmann et al., 2015). Aeruginosin-865 was the first variant found in the genus *Nostoc* and was isolated from the strain Lukešová 30/93 growing in forest soil (Kapuścik et al., 2013). Pseudoaeruginosins are a third group of similar linear peptides which contain mPro instead of Choi (Liu et al., 2015; Figure 2).

The non-ribosomal peptidome of *N. spumigena* typically includes nodularins, spumigins, aeruginosins, anabaenopeptins, and sometimes pseudoaeruginosins (Fewer et al., 2013; Mazur-Marzec et al., 2013; Liu et al., 2015). The corresponding biosynthetic gene clusters have been described from *N. spumigena* CCY9414 (Fewer et al., 2009, 2013; Voß et al., 2013).

In the current study we analyzed non-ribosomal peptides from nine *Nostoc* and nine other cyanobacterial genera that had been collected from Brazilian saline-alkaline lakes of the Nhecolândia, Pantanal wetland area. Our objective was to discover novel bioactive compounds. Surprisingly *Nostoc* sp. CENA543 produced NOD in quantities comparable to those produced by toxic *N. spumigena* strains that had been found in various geographical locations. A new peptide family pseudospumigins (Psp) expressed by *Nostoc* sp. CENA543 was identified (Table 1). The structure of Psps are identical with that of spumigins but the diagnostic amino acid Pro/mPro that are found in spumigins were replaced by Ile, Leu, or Val amino acids and hence the novel family were named the pseudospumigins. We found that the Psp family are trypsin inhibitors.

**Table 1:** *Nostoc* sp. CENA543 non-ribosomal peptides.

**No**.	**Nonribosomal peptide**	**[M+H]^+^*(m/z)***	**Δ (ppm)**	**Subunits**					**RI (%)**
				**1**	**2**	**3**	**4**	**5**	
1	Nodularin-R	825.4505	0.0	MeAsp	Arg	Adda	Glu	MeDhb	96
2	Desmethylnodularin-R	811.4349	−4.1	MeAsp/Asp	Arg	Adda	Glu	MeDhb/Dhb	4
3	Pseudospumigin A	613.3344	0.0	Hpla	D-Hty	L-Ile	Argininal	–	81
4	Pseudospumigin B	613.3344	−^a^	Hpla	Hty	Leu	Argininal	–	9
5	Pseudospumigin C	599.3188	−0.2	Hpla	Hty	Val	Argininal	–	5
6	Pseudospumigin D	597.3395	−2.1	Hpla	Hph	Ile	Argininal	–	4
7	Pseudospumigin E	597.3395	−^b^	Hpla	Hph	Leu	Argininal	–	<1
8	Pseudospumigin F	583.3239	−^b^	Hpla	Hph	Val	Argininal	–	<1

*[M+H]^+^ is the exact mass and Δ is the difference between the exact mass and the measured mass. RI, relative [M+H]^+^ intensity within the peptide group; Adda, 3-amino-9-methoxy-2,6,8-trimethyl-10-pheny1-4,6-decadienoic acid; MeDhb, N-methyl-didehydroaminobutyric acid; Hpla, p-OH-phenyllactic acid*.

a *Elute together with pseudospumigin A*.

b *Intensity too low*.

## Materials and methods

### Strains and cultivation

The cyanobacterial strains studied were isolated from water samples that had been collected from several saline-alkaline lakes located at Centenário farm in the southern part of the sub-region Nhecolândia in the north of the municipality of Aquidauana, Mato Grosso do Sul State, Brazil (Andreote et al., 2014; Genuário et al., 2017; Figure S1). The *Nostoc* sp. strains CENA543 and CENA544 were isolated from water samples collected in September 3, 2010 from the lake “Salina 67 Mil” (19°27′42″S, 56°08′21″W). An additional 7 *Nostoc* and 9 other cyanobacterial strains were also collected in May 27 or September 3, 2010 from the lakes “Salina 67 Mil,” “Salina Verde” (19°28′13″S, 56°03′22″W), “Salina Preta” (19°26′56″S, 56°07′55″W), “Salina Grande” (19°26′56″S, 56°07′45″W) and “Salina Centenário” (19°26′24″S, 56°05′58″W). The CENA543 strain was purified axenic before peptide analysis and DNA isolation. All strains were cultivated at ambient temperature (20–22°C), in continuous low photon irradiance (5–10 μE m^−2^ s^−1^), in low salinity (0.6‰) but at high phosphorus concentration (5,500 μg ${\text{PO}}_{4}^{3-}$-P L^−1^) on Z8 medium (Kotai, 1972) without a nitrogen source for a few weeks. The reference strain *N. spumigena* AV1 was cultivated as previously described (Fewer et al., 2009). The effect of 0, 1, 2, 3, 4, 5, and 6% salinities adjusted with NaCl to the growth of *Nostoc* sp. CENA543 was tested on Z8 medium. A 50 ml volume of medium was placed into 250 ml plastic cell cultivation flasks, each of which was inoculated with 1 ml of gently homogenized cell suspension that had been grown for 28 days on Z8 medium without a nitrogen source. Flasks were cultivated for 34 days in the horizontal position with 80 rpm shaking speed at 18°C. *Nostoc* sp. CENA543 was mass cultivated in 5 L Erlenmeyer flaks containing 2.7 L of Z8 medium with sterilized air bubbling for NMR, amino acid analysis and enzyme inhibition tests. The DNA extraction required to *Nostoc* sp. CENA543 be grown in 1% salinity for 16 days (addition of 8.75 g NaCl L^−1^ and 3.75 g MgSO_4_·7H_2_O L^−1^) in Z8 medium without a nitrogen source to reduce the slime formation, which made DNA isolation difficult.

### Peptide recognition by LC-MS

Cells were grown in 40 ml of liquid cultures, collected and freeze dried. The dried biomasses were placed in 2 ml plastic tubes together with 1 ml methanol and glass beads (0.5-mm diameter glass beads, Scientific Industries INC) and were shaken using FastPrep cell disrupter instrument three times for 30 s at a speed of 6.5 m·s^−1^. Tubes were then centrifuged 10,000 × *g* for 5 min at room temperature. Supernatants were analyzed first using low resolution LC-ESI-ITMS (Agilent 1100 Series LC/MSD Ion Trap XCT Plus, Agilent Technologies, Palo Alto, CA, USA). A 10 μl sample was injected into a Luna C18 column (2.1 × 100 mm, 5 μm, Phenomenex, Torrance, CA, USA) which was eluted by 30% acetonitrile (solvent B) in 0.1% HCOOH to 70% of B at 40°C for 49 min with a flow rate of 0.15 ml min^−1^. Mass spectra data were accumulated in ultrascan positive electrospray ionization mode (26,000 *m/z* s^−1^) at a scan range of *m/z* 300–2,200 and by calculating the mean of three spectra.

High resolution UPLC-QTOF analyses were performed using an Acquity I-Class UPLC—Synapt G2-Si HDMS (Waters Corp., Milford, MA, USA) system. Samples that ranged from 0.1 to 1 μl were injected into a Cortecs UPLC® C18+ column (2.1 × 50 mm, 1.6 μm, Waters), which was then eluted at 40°C with a flow rate of 0.3 ml min^−1^ from 10% acetonitrile (+0.1% HCOOH; solvent B) in 0.1% HCOOH to 70% of B in 5 min, then to 95% of B in 0.01 min, kept there 1.99 min, then back to 10% of B in 0.5 min and finally kept for 2.5 min before the next run. QTOF was calibrated using sodium formate, which gave a calibrated mass range from *m/z* 91.036 to 472.726 or from 1178.651 to 2121.195 depending of the run. Leucine Enkephalin was used at 10 s intervals as a lock mass reference compound. Mass spectral data were accumulated in positive electrospray ionization Resolution Mode at scan range of *m/z* 50–200 or 1,500–2,200 depending of the run.

### Pseudospumigin a purification

Pseudospumigin A was purified for NMR, amino acid analysis and the enzyme inhibition test. One gram of freeze dried *Nostoc* sp. CENA543 cells were lysed in a 25 ml volume of methanol in a 50 ml plastic tube for 30 s using a Silentcrusher M homogenizer (Heidolph Instruments GmbH & Co, Germany) at 20,000 rpm speed. After centrifugation 10,000 × *g* for 5 min at room temperature, the supernatant was removed and the extraction was repeated once with 20 ml of methanol. The combined methanol solutions were diluted with water to 80% methanol concentration. Solution was added into a methanol (20 ml) preconditioned solid phase C18-E cartridge (5 g/20 ml, Phenomenex, Torrance, CA, USA). The effluent was first evaporated by a vacuum rotatory evaporator and then freeze dried. The residue was dissolved in 15 ml methanol and 450 mg of NaBH_4_ was added to reduce the peptide aldehydes to chromatographically better behaving alcohols. After gas formation the methanol was vacuum rotatory evaporated. The residue was dissolved in 2 ml of eluent then used in pseudospumigins purification performed by an Agilent LC-ESI-ITMS. The solution was injected in 100–500 μl sample batches with a manual injector into a Luna C8(2) column (10 × 150 mm, 5 μm, Phenomenex, Torrance, CA, USA) eluted isocratically with solvent mixture of 1% aqueous ammonium acetate and acetonitrile (22:78) at speed of 5 ml min^−1^. Collected fractions were pooled then diluted in 1:2 with water and concentrated with preconditioned (first 6 ml methanol then 6 ml water) solid phase Strata-X cartridge (1 g/6 ml, Phenomenex). The cartridge was first washed with 6 ml water which was then blown out with compressed air and pseudospumigins (Psp A fraction) were eluted from the cartridge with 3 ml of methanol yielding 0.87 mg of freeze dried solid material.

### NMR and UV spectroscopy

NMR spectra for structural elucidation of pseudospumigin A were collected using an Avance III HD 500 MHz NMR spectrometer equipped with ^1^H, ^13^C, ^15^N TXI probe and an Avance III HD 800 MHz NMR spectrometer equipped with ^1^H, ^13^C, ^15^N TCI cryoprobe. All spectra were collected at room temperature. The COSY experiment at 500 MHz were obtained with 8 transients, using 256 and 1,024 complex points in t_1_ and t_2_ domains that corresponded to acquisition times of 46.6 and 186.2 ms, respectively. At 800 MHz, the ^1^H spectrum was collected using 16 transients and 16 k complex points that corresponded to an acquisition time of 1.278 s. The sensitivity enhanced ^13^C-HSQC (^15^N-HSQC) spectrum was obtained using 128^32^ and 1,024 complex points in t_1_ (t_1_) and t_2_ dimensions. This results in acquisition times of 3.53 ms (1.6 ms) and 79.9 ms in ^13^C (^15^N) and ^1^H dimensions, respectively. The signal was accumulated with 32 (160) transients per FID in ^13^C-HSQC (^15^N-HSQC) experiment. The data for heteronuclear multiple bond correlation (^13^C-HMBC) spectrum was collected with 128 and 2,048 complex points in t_1_ and t_2_ domains, using 160 transients per FID. This translates to acquisition times of 2.9 and 213 ms in ^13^C and ^1^H dimensions, respectively. The long-range ^1^H-^13^C transfer delay was optimized according to 6 Hz ^n^J_CH_ couplings. UV (MeOH) λ_max_ (log ε) 225 (4.23), 278 (3.49) nm.

### Nodularin-R quantitation

A 10.14 mg quantity of freeze dried *Nostoc* sp. CENA543 and a 12.45 mg of freeze dried sample of *N. spumigena* AV1 cells were extracted with 1 ml methanol as previously explained. A 50-fold methanol diluted extracts together with eight nodularin a (a gift from Z. Grzonka, University of Gdansk, Gdansk, Poland) standard methanol solutions (2-fold dilutions from concentration 2 to 0.0156 μg ml^−1^) were analyzed by UPLC-QTOF using negative ionization. Nodularin peak areas of *m/z* 823.432 ± 0.01 ion chromatograms were measured. Nodularin gave a standard curve y = 1010x^2^ + 2540x − 58.6 (y = peak area, x = nodularin concentration) with *R*^2^ = 0.9999 from which the *Nostoc* sp. CENA543 and *N. spumigena* AV1 nodularin concentrations were calculated.

### Amino acid analysis

A 50 μg quantity of purified Psp A fraction in a 200 μl glass tube inside a closed 4 ml vial containing 500 μl of 6 M HCl was hydrolyzed at 110°C for 12 h. The glass tube content was vacuum dried and 50 μl of water, 20 μl of 1 M NaHCO_3_, and 100 μl of 1% Marfey reagent in acetone (1-fluoro-2,4-dinitrophenyl-5-L-alanine or L-leucine amide) was added. The reaction was stopped after 1 h of incubation at 37°C by adding 20 μl of 1 M HCl and the solution was analyzed by UPLC-QTOF. The reference compounds from which L- and D-Leu, L-, D-, and L-*allo*-Ile, L- and D-Val and DL-Hph were obtained from Sigma-Aldrich (Switzerland), whereas L- and D-Hty were obtained from ABCR GmbH & Co. (Germany) and were Marfey derivatized accordingly.

### IC_50_ measurement

Trypsin (porcine pancreas, Sigma-Aldrich) activity was measured at 25°C in a reaction mixture that contained from 0 to 81.6 mM leupeptin or pseudospumigin A and the hydrolysis was followed at every 2 min for 1 h, as described earlier (Liu et al., 2015).

### DNA extraction and genome sequencing

Cells were collected in a 50 ml centrifuge tube and centrifuged at 6,000 × *g* for 5 min. Cells were washed with 2 ml of Tris-EDTA-NaCl-buffer (50 mM Tris-HCl—100 mM EDTA—100 mM NaCl) and transferred to microcentrifuge tubes. The tubes were centrifuged at 7,000 × *g* for 4 min and the supernatant was discarded. Glass beads were added to the tubes and the cells were frozen at −80°C. Samples were thawed at 64°C and 800 μl of GOS-buffer (100 mM TrisHCl, pH 8, 1.5% SDS, 10 mM EDTA, 1% deoxycholate, 1% Igepal-CA630, 5 mM thiourea, 10 mM dithiothreitol; Kolmonen et al., 2004) was added to the cells. The cells were then disrupted using FastPrep at 5 m.s^−1^ for 20 s. The samples were kept on ice for 5 min and centrifuged at 15,000 × *g* for 1 min. The supernatant was pipetted in a 15 ml centrifuge tube. The disruption of the cells and centrifugation steps were repeated with additional 800 μl of GOS-buffer. The supernatants were combined by decanting into the 15 ml centrifuge tube. A volume of 225 μl of 5 M NaCl water solution and 170 μl of 10% CTAB (10% CTAB–0.7 M NaCl) was added to each 800 μl of buffer. The sample was then divided amongst four tubes, each of which was mixed and incubated at 65°C for 20 min. An equal volume of phenol-chloroform (1:1) was added. Samples were mixed and centrifuged at 10,000 × *g* for 7 min. The water phases that contained the DNA was transferred into fresh tubes and phenol-chloroform treatment was repeated. The isopropanol (5 mL) was added to the tubes, which were mixed and centrifuged at 10,000 × *g* for 15 min at 4°C. The supernatant was discarded and the pellet was dissolved in 1 ml of 70% ethanol. The samples were combined and centrifuged at 10,000 × *g* for 5 min. The supernatant was discarded and the samples were dried at 37°C and stored at −20°C.

The DNA pellet was dissolved in 850 μl of TE-buffer and 1.6 μl of RNAse A was added. Each sample was incubated at 37°C for 30 min. A saline solution of 5 M NaCl was added to a final concentration of 0.2 M (32 μl). An equal volume of phenol-chloroform was added and the sample was centrifuged at 20,000 × *g* for 5 min. The water phase that contained the DNA was transferred into a new tube and an equal volume of chloroform was added and the sample was centrifuged at 16,000 × *g* for 5 min. The DNA sample was transferred to a new tube and an equal volume of isopropanol was added and centrifuged at 10,000 × *g* at 4°C for 10 min. The supernatant was discarded and the pellet was washed with 70% ethanol followed by drying at 37°C. The DNA pellet was dissolved in 300 μl of TE-buffer. The purity, concentration and quality of the DNA was measured and evaluated using Nanodrop ND-1000 Spectrophotometer (Nanodrop Technologies, USA), gel electrophoresis and an Agilent TapeStation (Agilent Technologies). High-molecular DNA was subjected to library (Illumina TruSeq® PCR Free 350 bp) construction and sequenced by using an Illumina HiSeq2500 platform with a paired ends 100 cycles run. The gaps of the gene cluster were closed by PCR and Sanger sequencing.

### Biosynthetic gene cluster analysis

The genome data (1 Gb) were first checked by the Spades software (version 3.7.1) for read correction and removal of erroneous readings, and then the data were assembled using the Newbler program (version 3.0). The genome sequence obtained for *Nostoc* sp. CENA543 was analyzed using antiSMASH (Blin et al., 2017) and annotated using Artemis software (Rutherford et al., 2000). The sequence was analyzed for the NRPS/PKS content using PKS/NRPS Analysis (Bachmann and Ravel, 2009) and the substrate prediction of the adenylation domain was done using NRPS predictor 2 (Rausch et al., 2005; Röttig et al., 2011) and manual alignment. The phylogenetic analysis was performed in the Molecular Evolutionary Genetics Analysis (MEGA 6.06; Tamura et al., 2013). A phylogenetic tree was constructed using Neighbor-joining (16S rRNA genes—K2+G, *ndaF* gene—K2+G, SpuA and SpuB amino acids—Poisson model +G) and Maximum likelihood (16S rRNA genes—K2+G+I) methods.

## Results

### Growth of *Nostoc* sp. CENA 543 and identification of peptides

We studied the non-ribosomal peptides of the cyanobacterium *Nostoc* sp. CENA543 isolated from a saline-alkaline lake in the Pantanal wetland area of Brazil (Figure S1). A growth experiment demonstrated that *Nostoc* sp. CENA543 grew best at low (0.6‰) salt concentration despite, some occasionally high salt concentrations (4.5%) that were encountered in the lake (Figure S1). Growth at 1% salinity was 10 times slower and growth ceased completely at 2% salinity. Therefore, we cultivated this strain in low salinity and light but at high phosphorus concentrations in order to obtain good growth and peptide expression. *Nostoc* sp. CENA543 grew on culture flask surfaces and showed a benthic growth profile (Figure S1).

**Figure 3:**
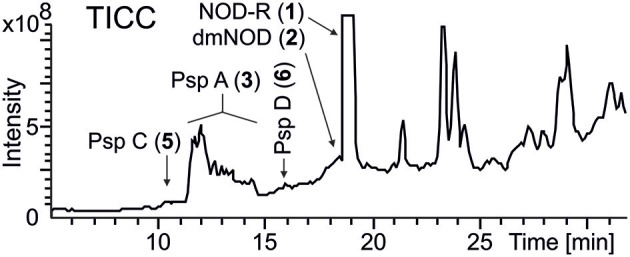
LC-ITMS total ion current chromatogram (TICC) of *Nostoc* sp. CENA543. Abbreviations as in Figure 1.

A methanol extract of the culture was first analyzed by high performance liquid chromatography electro-spray ion trap mass spectrometry (HPLC-ITMS), which resulted to the identification of hepatotoxic nodularin-R in high quantities and lower quantities of desmethylnodularin-R (Figure 3, Figure S2). Quantitative analysis performed by using ultra performance liquid chromatography guadrupole time of flight (UPLC-QTOF) mass spectrometry determined that *Nostoc* sp. CENA543 produced 4.3 mg NOD g^−1^ biomass DW (20–22°C, 5–10 μE m^−2^ s^−1^, salinity 0.6‰, 5,500 μg PO_4_-P L^−1^), which was more than twice as much NOD production by *N. spumigena* AV1 (1.9 mg g^−1^). The chemical structures of the nodularin variants were assigned using the product ion spectra generated by UPLC-QTOF and HPLC-ITMS (Figure S2).

**Figure 4:**
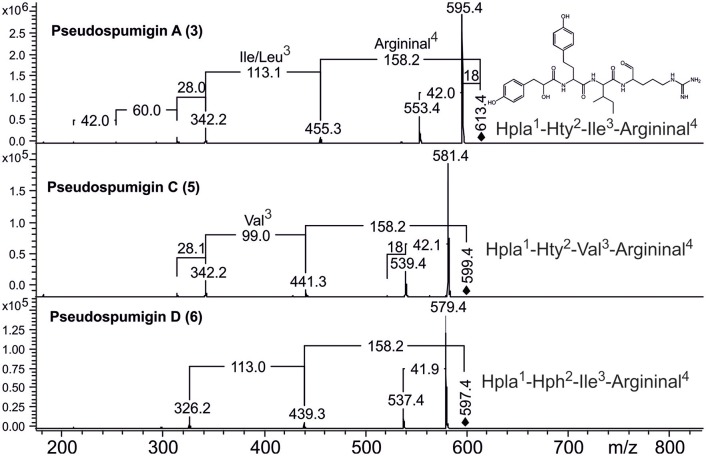
Product ion spectra and structures of pseudospumigins A (**3**), C (**5**), and D (**6**) identified from *Nostoc* sp. CENA543.

**Figure 5:**
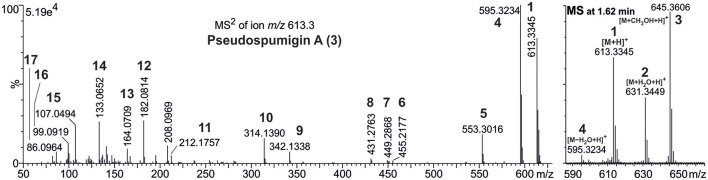
Accurate mass product ion spectrum (MS^2^) of protonated pseudospumigin (Psp) A (**3**) *m/z* 613.3 and mass spectrum (MS) from the chromatographic peak of Psp A identified from *Nostoc* sp. CENA543. Numbers in bold refer to the ion structures presented in Table 1.

HPLC-ITMS analysis of the methanol extract also revealed that there was an early eluting compound group whose chromatographic behavior (broad tailing peaks) and MS fragmentation resembled those of the spumigin and aeruginosin aldehydes (Figure S2; Fewer et al., 2009, 2013). Analysis of the low and high resolution spectra showed that these pseudospumigins (Psps) are closely related to spumigins and aeruginosins that have Pro/mPro or Choi diagnostic amino acids but differ from them by the substitution with Ile/Leu or Val amino acids (Figures 2, 4, 5). Ion assignments of the high-resolution product ion spectrum of protonated Psp A, C, D, and F are presented in Table 2, Table S1, and in Figure S3. The subunit sequence of the Psp family was found to be Hpla^1^-Hty/Hph^2^-Ile/Leu/Val^3^-Argininal^4^. The aldehyde group of Psp's was seen also in the formation of hydrate (*m/z* 631.34 [M+H]^+^ for Psp A) and hemiacetal (*m/z* 645.36 [M+H]^+^ for Psp A) structures (Figure 5). Argininol^4^ containing variants were practically absent. Chiral amino acid analysis of the isolated Psp mixture revealed the amino acids D-Hty, D-Hph, L-Ile, L-Leu, and L-Val, although L-Hph was also detected (Figure S4). This finding together with the ^1^H-^13^C HSQC spectrum analysis showed that roughly one tenth of the Aa^3^ was L-Leu. This finding indicates that Hpla^1^-Hty^2^-Leu^3^-Argininal^4^ (Psp B) and Hpla^1^-Hph^2^-Leu^3^-Argininal^4^ (Psp E) variants were also present in the sample. The Hpla^1^-Hph^2^-Val^3^-Argininal^4^ variant (583 Da, Psp F) was detected only by the UPLC-QTOF analysis. The NMR data that are presented in Supplementary Material (Figure S5, Table S2) were in line with the published data from Sps, aeruginosins, and nostosins and confirmed the Psp structure. The relative amounts of the Psp entities that were calculated from the protonated Psp HPLC-ITMS peaks and NMR data are presented in Table 1.

**Table 2:** Ion assignments, calculated, and measured ion masses and mass difference (Δ) of protonated pseudospumigin A (**3**) isolated from *Nostoc* sp. CENA543 analyzed by UPLC-QTOF.

**No**.	**Ion assignment**	**Calculated**	**Measured**	**Δ (ppm)**
1	M + H^+^	613.3344	613.3345	0.1
2	M + H_2_O + H^+^	631.3450	631.3449	−0.2
3	M + CH_3_OH + H^+^	645.3606	645.3606	−0.1
4	M – H_2_O + H^+^	595.3239	595.3234	−0.9
5	M – (HN=C=NH + H_2_O) + H^+^	553.3021	553.3016	−0.9
6	Hpla-Hty-Ile + H^+^	455.2177	455.2177	0.0
7	Hty-Ile-Argininal + H^+^	449.2871	449.2868	−0.7
8	Hty-Ile-Argininal – H_2_O + H^+^	431.2765	431.2763	−0.6
9	Hpla-Hty + H^+^	342.1336	342.1338	0.4
10	Hpla-Hty – CO + H^+^	314.1387	314.1390	0.8
11	Ile-Argininal – (HN=C=NH + H_2_O) + H^+^	212.1757	212.1757	−0.4
12	Hpla-NH_2_ + H^+^	182.0812	182.0814	1.0
13	Hpla-NH_2_ – H_2_O + H^+^	164.0706	164.0709	1.5
14	Hty → HO-C_6_H_4_-C_3_H_3_ + H^+^	133.0648	133.0652	2.7
15	HO-C_6_H_4_-CH_2_ + H^+^	107.0491	107.0494	1.9
16	Argininal → C_5_H_11_${\text{N}}_{2}^{+}$	99.0917	99.0919	1.8
17	Ile – CO + H^+^	86.0964	86.0964	−0.9

**Figure 6:**
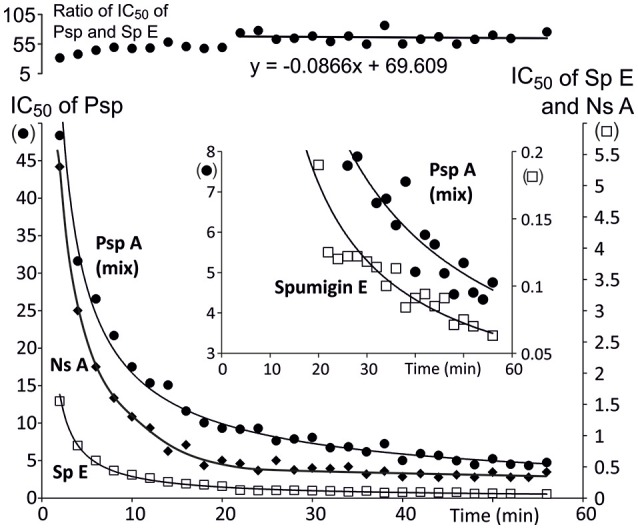
Time dependence of trypsin inhibition efficiency (IC_50_) of pseudospumigin A [•, Psp A (**3**) + other Psp variants], nostosin A (♦, Ns A) and spumigin E (□, Sp A, from Fewer et al., 2013). The uppermost curve shows that the ratio of IC_50_ of Psp mix and Sp E stabilizes and that trypsin inhibition by Psp mix is 1/70th the inhibition of spumigin E. The insert is an enlargement from the small IC_50_ values of Psp mix and Sp E clearly shows that pseudospumigin A (mix) and spumigin E trypsin interaction were not in equilibrium after 1 h of reaction time.

The bioactivity of the isolated and purified Psp mixture from which about 80% is Psp A was tested with trypsin because many structurally analogous Sps, aeruginosins, pseudoaeruginosins, and the more distant nostosins homologs are all trypsin inhibitors. Psp A (mix) inhibited trypsin in a time dependent manner as Sp E^15^ (Figure 6). When trypsin activity was measured for different Psp A (mix) concentrations at 1 h, the IC_50_ value of Psp A (mix) was 4.5 μM. At that point the trypsin inhibition activity of Psp was 1/70th the activity of Sp E.

Both *Nostoc* strains of CENA543 and CENA544 that had been isolated from lake “Salina 67 Mil” had identical peptide profiles, whereas the third Salina 67 Mil sample, which was a non-*Nostoc* strain had a different profile. Moreover, all the other 18 *Nostoc* and non-*Nostoc* strains that had been collected from the nearby Nhecolândia Pantanal wetland lakes presented different peptidomes (Figures S6, S7).

### *Nostoc* sp. CENA 543 biosynthetic gene clusters

**Figure 7:**
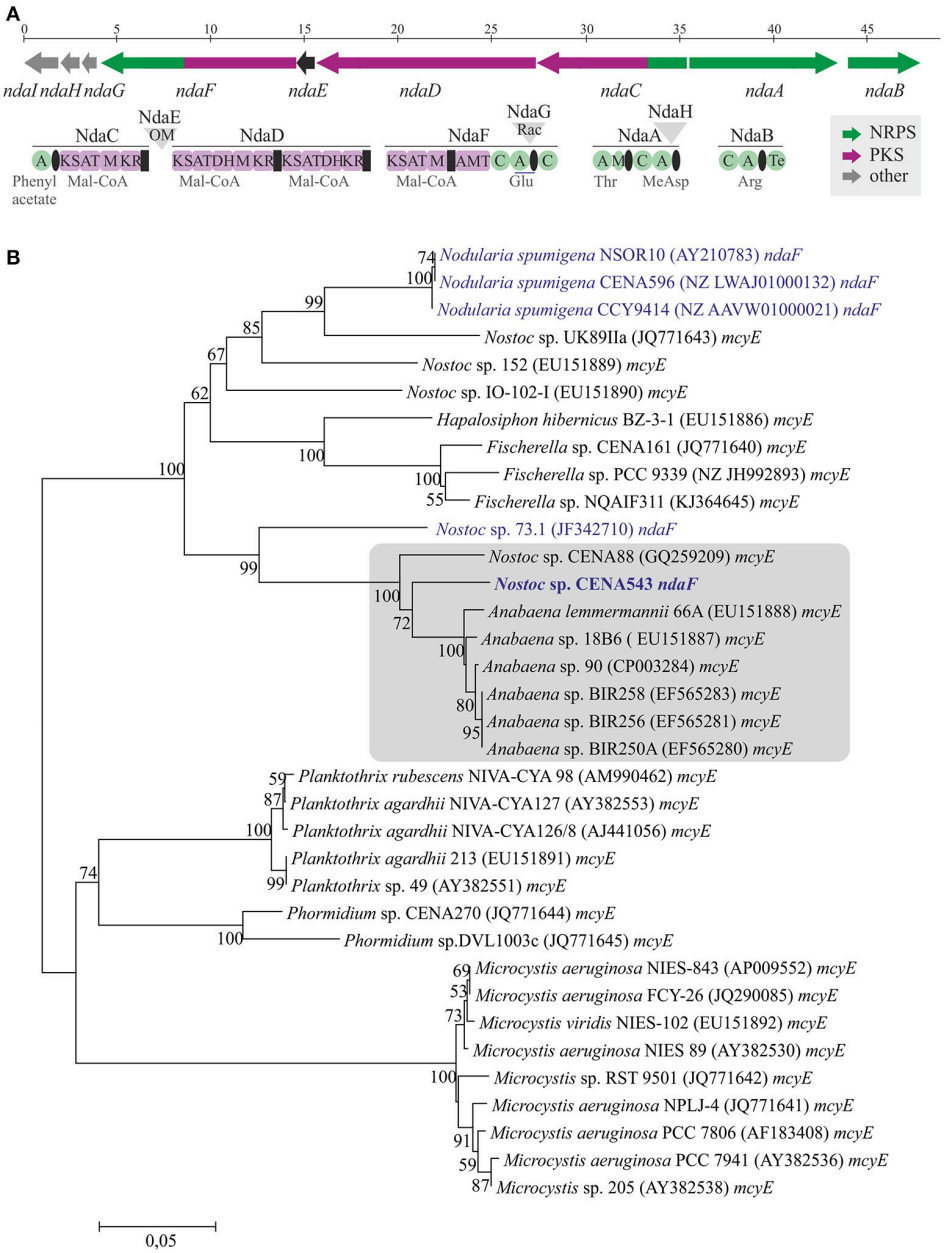
Nodularin biosynthetic gene cluster from *Nostoc* sp. CENA543 **(A)** and evolutionary history of *ndaF* and *mcyE* genes **(B)**. Neighbor-joining tree based on *ndaF* and *mcyE* genes in the adenylation domain and peptidyl carrier protein region. Bootstrap values above 50 percent of 1,000 replicates are given at the nodes. Strains that produce nodularin are highlighted in blue and *Nostoc* sp. CENA543 is blue and bold.

A 7.2 Mb draft genome sequence organized in 67 contigs was obtained from the *Nostoc* sp. CENA543 strain (data not shown). A complete 48 kb NOD biosynthetic gene cluster that encode hybrid NRPS-PKS enzymes was identified in the draft genome (Figure 7A). The *Nostoc* sp. CENA543 NOD biosynthetic gene cluster (GenBank accession number MF668122) was found to be similar to the NOD biosynthetic gene clusters of *Nostoc* sp. 73.1 and microcystin gene cluster of *Anabaena* sp. 90 (Table S3). The nodularin gene cluster of *Nostoc* sp. CENA543 comprised two NRPSs (NdaA and NdaB), two hybrid NRPS-PKS (NdaC and NdaF), a PKS (NdaD), an O-methyltransferase (NdaE), a racemase (NdaG), a dehydrogenase (NdaH), and an ABC transporter (NdaI; Table S3).

**Figure 8:**
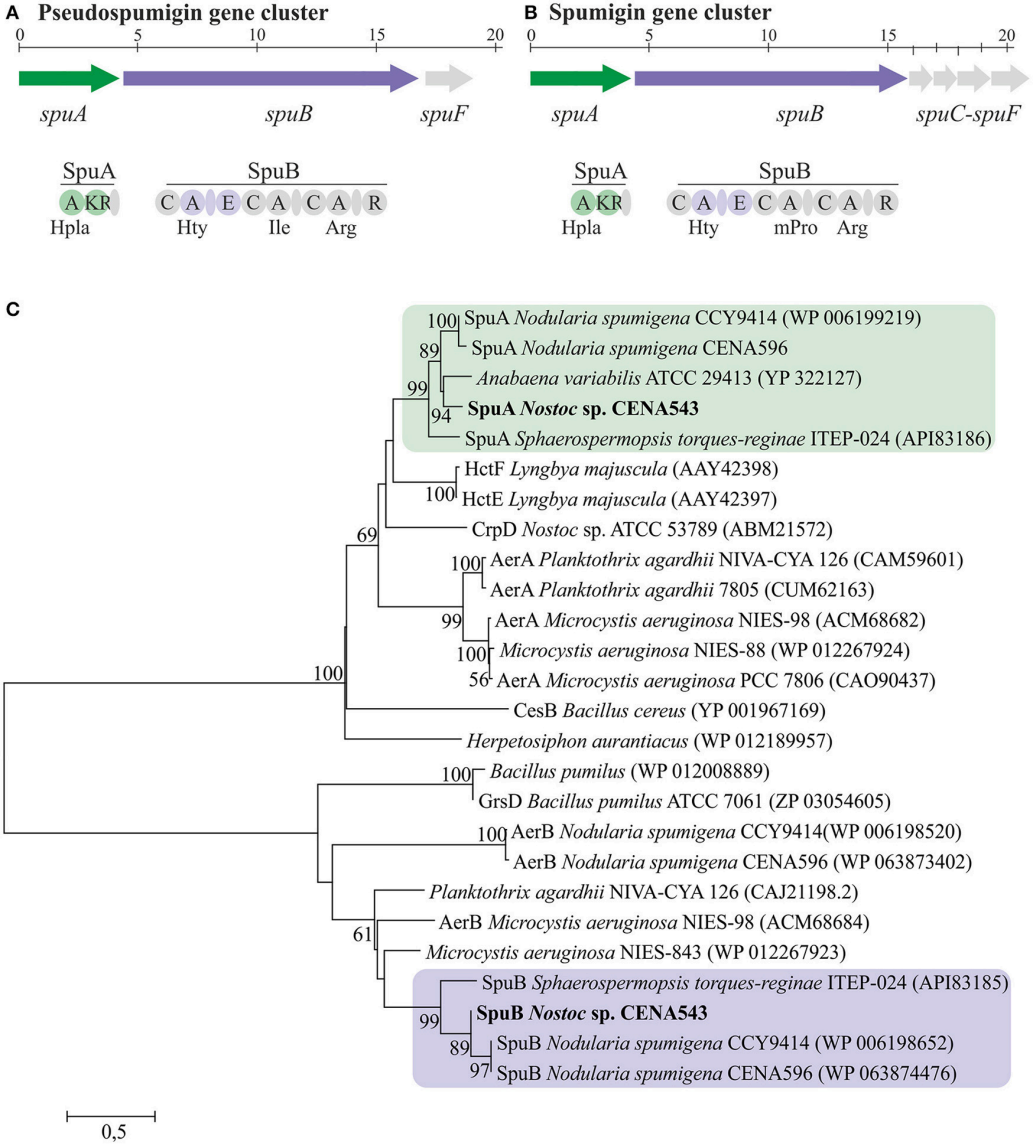
The evolutionary history of SpuA and SpuB of pseudospumigin. Pseudospumigin gene cluster of *Nostoc* sp. CENA543 **(A)**, Spumigin gene cluster of *Nodularia spumigena* CCY9414 **(B)**, and phylogenetic (neighbor-joining) tree based on SpuA and SpuB amino acid sequences **(C)**. Bootstrap values above 50 percent of 1,000 replicates are given at the nodes. *Nostoc* sp. CENA543 is highlighted and region of the proteins used for the analyses are colored in green or purple **(A,B)**.

The complete 19 kb Psp biosynthetic gene cluster was located through BLASTp search using spumigin biosynthetic enzymes as the query (Figure 8A and Table S3, GenBank accession number MF668123). The Psp biosynthetic gene cluster encodes two NRPS enzymes, SpuA, and SpuB in addition to the ABC transporter, SpuC (Figure 8A, Table S3). The amino acids predicted to be incorporated by the adenylation domains in the NRPS of nodularin and pseudospumigin were found to be partly consistent with the chemical structures obtained from these compounds (Table S4).

### Evolutionary history of *Nostoc* sp. CENA543 and non-ribosomal biosynthetic genes

**Figure 9:**
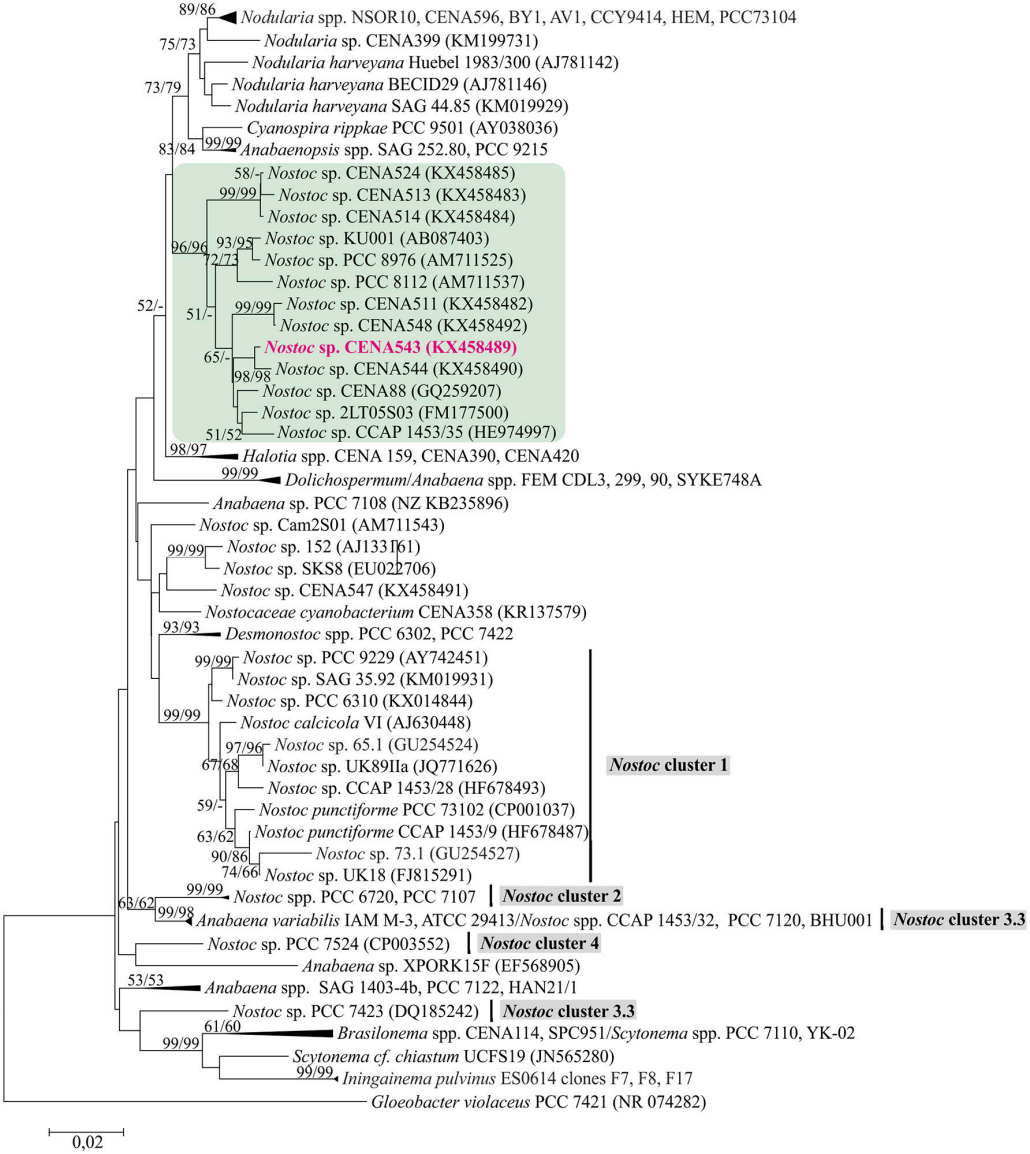
Phylogenetic analysis of 16S rRNA gene including *Nostoc* sp. CENA543. Neighbor-joining tree based on 16S rRNA gene. Bootstrap values above 50 percent from 1,000 replicates are given at the nodes (Neighbor-joining/Maximum likelihood). *Nostoc* sp. CENA543 is highlighted in pink.

The phylogenetic tree that was constructed using 16S rRNA gene sequences show that *Nostoc* sp. CENA543 grouped with other *Nostoc* strains, which are more closely related to *Nodularia, Anabaenopsis*/*Cyanospira*, and *Halotia* strains than with other *Nostoc* strains (Figure 9). Nodularin producers are widely distributed in this phylogenetic tree according to their taxonomy. A phylogeny based on *ndaF* and *mcyE* genes from nodularin and microcystin biosynthetic gene clusters, respectively, indicate the multiple origin of nodularin producers (Figure 7B). Interestingly, the *ndaF* gene of *Nostoc* sp. CENA543 was closely related to *mcyE* genes of the strains that produce microcystins. The evolutionary history of SpuA and SpuB proteins involved in the synthesis of Psps in *Nostoc* sp. CENA543 shows a high similarity with the spumigin gene cluster (Figure 8C, Table S3).

## Discussion

We studied the peptidomes of nine Brazilian Pantanal wetland extremophilic *Nostoc* and nine other strains of cyanobacterial genera, which have been isolated from saline-alkaline lakes. The results indicated that *Nostoc* spp. CENA543 and CENA544 have identical peptide profiles that diverged from the profiles of the other analyzed strains and results concerning nodularins and pseudospumigins are reported herein. *Nostoc* sp. CENA543 isolated from a Brazilian, shallow, saline-alkaline lake water is a free-living cyanobacterium that under laboratory conditions showed benthic growth (Figure S1). Nodularin-R (NOD-R) was the predominant NOD variant detected (Table 1). The production of NOD by the *Nostoc* sp. CENA543 was shown both at the genetic and the metabolic levels. NOD was originally found in the genus *Nodularia*, whose representatives originate mostly from brackish waters but are more rarely found in fresh water (Beattie et al., 2000; Akcaalan et al., 2009; Sivonen, 2009). Motuporin and isomotuporins are analogous compounds to nodularin, which contain Val, Ile, or 2-aminobutyric acid instead of Arg in position 2 and Adda or demethoxyAdda in position 3, have been reported in marine sponge *Theonella swinhoei* (de Silva et al., 1992; Figure 1). It has been commonly suggested that associated cyanobacteria are actually responsible for the production of motuporins (de Silva et al., 1992). Several cyanobacterial genera including *Nostoc* produce microcystins, which are close structural relatives to NODs (Figure 1). The finding of NOD synthesis by cycad and lichen symbiotic *Nostoc* strains was the first direct evidence of NOD production by another cyanobacterial genus than *Nodularia* (Gehringer et al., 2012; Kaasalainen et al., 2012). Recent studies reported that NODs were identified from a symbiotic *Nostoc* of liverwort *Blasia pusilla* L, and also from a new cyanobacterium genus and species *I. pulvinus* gen nov., sp. nov. that was found in a freshwater wetland spring (Liaimer et al., 2016; McGregor and Sendall, 2017). NOD-R has been previously identified from benthic cyanobacterial mats found in Lake Tikitapu, New Zealand, which contained *Nostoc* and lacked *Nodularia* strains (Wood et al., 2012). However, none of the isolated strains produced NOD-R, which in the light of what we found in the present study suggests that a non-isolated *Nostoc* strain might have been the NOD-R producer (Wood et al., 2012).

Quantitative UPLC-QTOF analysis showed that in low light, low salinity and high phosphorus environment without further optimization, laboratory cultivated CENA543 strain produced 4.3 mg NOD g biomass DW. This NOD production level is high and at the same level as that found in planktonic *N. spumigena* and benthic *N. sphaerocarpa* strains but more than four orders of magnitude higher compared to laboratory grown cycad endosymbiotic *Nostoc* strains (Table 3). In symbiosis NOD levels were 0.0025 μg g^−1^ in cycads and <10–60 μg g^−1^ in lichens. The NOD levels of lichens were thus four orders of magnitude higher than those expressed in cycads. About 7% of the lichen biomass is photobiont (Ahmadjian, 1993), which gives <0.1–0.9 mg NOD g^−1^ cyanobiont in lichens. Consequently, the rough estimate is that in lichens the cyanobiont, which most probably is *Nostoc*, will produce one tenth of the NOD levels of free living *Nostoc* sp. CENA543. It is, therefore, possible that lichens are much more resistant against NOD than are cycads. The resistance of lichens to hepatotoxins has already been reported earlier for microcystins (Kaasalainen et al., 2012). The mean level of NOD produced by the cyanobacterium *I. pulvinus* was 0.9 mg g^−1^ biomass DW, which is at the low level of *N. spumigena* NOD production but one fifth that of *Nostoc* sp. CENA543 NOD production (Table 3).

**Table 3:** Nodularin (NOD) concentrations (mg NOD/g dry weight) in laboratory grown *Nostoc* and *Nodularia* strains, *Nodularia* blooms and in cycad and lichens.

**Year(s)^a^**	**Location, strain**	**mg/g**	**References**
**LABORATORY GROWN STRAINS**			
2010	Brazil, Lake Salina 67 Mil, *Nostoc* sp. CENA543	4.3	This study
–	Australia, cycad, *Nostoc* sp. *riedlei* 65.1′	0.00035^b^	Gehringer et al., 2012
–	Australia, cycad, *Nostoc* sp. *serpentine* 73.1′	0.00023^b^	Gehringer et al., 2012
1987	Baltic Sea, *N. spumigena* AV1	1.9	This study
1966	France, Freshwater, *N. sphaerocarpa* PCC7804	1.2^c^	Saito et al., 2001
1966	France, Freshwater, *N. sphaerocarpa* PCC7804	4.2^c^	Beattie et al., 2000
1987	Baltic Sea, *N. spumigena* AV1	2.3	Fujii et al., 1997
1986	Baltic Sea, Arkona Basin, *N. spumigena* BY1	4–16	Lehtimäki et al., 1997
1985–1987	Baltic Sea, *Nodularia spumigena*	2.5–8.0	Sivonen et al., 1989
**BLOOMS**			
1994–2005	Baltic Sea, Gulf of Gdansk	0.1–4.0	Mazur-Marzec et al., 2006
1994	Lake Zeekoevlei, South Africa	3.5	Harding et al., 1995
1992–1993	Orielton Lagoon, Tasmania	2.0–3.5	Jones et al., 1994
1990–1991	Baltic Sea, Bothnian Sea	0.3–18.1	Kononen et al., 1993
1985–1987	Baltic Sea	0.1–2.4	Sivonen et al., 1989
**CYCADS AND LICHENS**		μ**g/g**	
–	Australia, cycad *Nostoc* symbiont	0.0025^b^	Gehringer et al., 2012
2009	Lichen talli	<10–60	Kaasalainen et al., 2012

a *Sample collection year(s)*.

b *mg NOD/g wet weight*.

c *[Har^2^]NOD*.

A new peptide family in the *Nostoc* sp. CENA543 was identified, which was named pseudospumigins (Psp; Figure 2) due to the close genetic and chemical structure resemblance to those of the spumigins. Spumigins have been found in *N. spumigena, A. compacta* NIES-835 and *S. torques-reginae* ITEP-024 strains (Fujii et al., 1997; Anas et al., 2012; Sanz et al., 2015). The spumigins structurally belong to the linear four subunit peptides, the diagnostic amino acid is Pro/mPro in position 3, whereas in the pseudospumigins this amino acid is absent and replaced by Ile, Leu, or Val. Furthermore, the genes that encode mPro in spumigin biosynthesis (Fewer et al., 2009) were absent from the pseudospumigin gene cluster. The aeruginosins are also structurally closely related to the pseudospumigins and the spumigins but the diagnostic amino acid is Choi (Figure 2). Many cyanobacterial genera produce aeruginosins but the aeruginosin family has been described in only three *Nostoc* strains: i.e., the terrestrial Lukešová 30/93 strain and recently found in plant leaves originating CENA352 and 458 strains (Kapuścik et al., 2013; Sanz et al., 2015). Nostosins produced by terrestrial *Nostoc* sp. FSN are homologous with aforementioned peptide groups but one of the middle amino acids is missing (Liu et al., 2014). In total well over 100 different structures that represent these peptide groups have been described. The *Nostoc* sp. CENA543 strain is not only excellent NOD producer, which is a typical feature for the *Nodularia* strains, but also produce pseudospumigins, which are very near structural analogs of spumigins and are almost exclusively known to be produced by *N. spumigena* strains. The pseudospumigin production level by the *Nostoc* sp. CENA543 was 0.87 mg purified from one g of biomass DW, which is roughly half that of the spumigin production level found in *N. spumigena* AV1 (Fewer et al., 2009) for similar purification yields. This result also links the peptidomes of *Nostoc* sp. CENA543 and *N. spumigena* strains more closely together. However, aeruginosins/pseudoaeruginosins (Figure 2) produced by the *N. spumigena* strains (Fewer et al., 2013; Mazur-Marzec et al., 2013; Liu et al., 2015) were not found in the *Nostoc* sp. CENA543 strain. Aeruginosins are estimated to be produced at significant amounts by *N. spumigena* AV1, 3 mg g^−1^ biomass DW (Fewer et al., 2013; Liu et al., 2015).

The Psp mixture (80% Psp A) was a trypsin inhibitor as are the structural analogous Sps, aeruginosins, and nostosins, which all contain a quanidino group in the Arg derivative substructure (Supporting Information in Liu et al., 2014). However, there was a wide variation in the IC_50_ values (μM) from 0.037 (chlorodysinosin A) to 94 (aeruginosin 126A) of these compounds without there being any clear association with the chemical structures. The inhibition by Psp A (mix) increased until the end of the 1 h measurement period as was the case for spumigin E (Figure 6). Time dependent inhibition is seen as a slow, tight and irreversible binding by inhibitors (Copeland, 2005). At the latter half of the measurement the ratio of IC_50_ of Psp A and Sp E stabilizes so that the Psp A (mix) was roughly 1/70th the strength of Sp E as a trypsin inhibitor and at the end of the measurement period the IC_50_ value was 4.5 μM. This large difference in the IC_50_ values was unexpected as the structures of Psp A and Sp E (Figure 2) of the subunits 1, 2, and 4 are identical. The fourth subunit of both compounds are also equal size and hydrophobically related amino acids, Ile, or Pro. The major difference between Psp A and Sp E is that the cyclic structure of the proline side chain makes Pro structurally rigid, which may in turn, affect the formation/dissociation of the enzyme-inhibitor complex. Psp A and nostosin A have identical subunits argininal and Ile but in nostosin A there is a 2-hydroxy-4-(4-hydroxyphenyl)-butanoic acid, one methylene unit longer Hpla analog and there is no fourth subunit. Nostosin A trypsin inhibition was also time dependent at the beginning of the measurement, but after 40 min IC_50_ stabilizes at a value of 0.35 μM (Liu et al., 2014; Figure 6). Trypsin inhibition by nostosin A was found to be 10-fold that of Psp A (mix). The commercial trypsin inhibitor, leupeptin, has the structure Ac-Leu-Leu-Argininal that resembles Psp A, Sp E and also nostosin A, and it interacts with trypsin without time dependency (Aoyagi et al., 1969; Kurinov and Harrison, 1996). The reasons for how these compounds show such different interactions with trypsin despite having lot of structural similarity remains to be investigated.

Morphological evaluations have shown that the cyanobacterial strain CENA543 belong to the genera *Nostoc* (Genuário et al., 2017). In a phylogenetic tree based on 16S rRNA (Figure 9), *Nostoc* sp. CENA543 and other *Nostoc* strains that were collected from the same Pantanal wetland area grouped together in a *Nostoc*-like cluster. *Nostoc*-like strains are more closely related to the *Nodularia* clade than to the true *Nostoc* clade (Herdman et al., 2015), in which the microcystin producing lichen symbiotic *Nostoc* sp. UK18 and UK89IIa were found (Figure 9). A *Nostoc*-like cluster is formed by sequences generated from free living cyanobacteria, such as the strain collected from a laundromat discharge pond in Michigan, USA (*Nostoc* sp. PCC 8112), or from brackish marshland on the Mediterranean coast of France (*Nostoc* sp. PCC 8676), or from a non-flooded rice field in Thailand (*N. elgonense* TH3S05), or from Brazilian mangroves (*Nostoc* sp. CENA175) or from a freshwater reservoir in Brazil (*Nostoc* sp. CENA88) (Genuário et al., 2017).

The evolutionary history based on *ndaF* and *mcyE* gene sequences indicates that different evolutionary events were responsible for the existence of nodularin producers (Figure 7B). Interestingly, *ndaF* of the *Nostoc* sp. CENA543 was more closely related to *Anabaena* spp. and to the microcystin producers of *Nostoc* sp. than to the nodularin producer *Nostoc* sp. 73.1 (Figure 7B). Previous studies indicate that the nodularin gene cluster has evolved after a deletion event in the microcystin gene cluster (Moffitt and Neilan, 2004; Rantala et al., 2004; Gehringer et al., 2012). Our analysis is consistent with this hypothesis. The biosynthetic gene cluster of pseudospumigin of the *Nostoc* sp. CENA543 is very similar to previously described spumigin gene cluster (Fewer et al., 2009; Figure 8). The phylogenetic analysis that used SpuA and SpuB amino acid sequences also indicated the closer relation of the pseudospumigin and spumigin sequences (Figure 8C).

## Author contributions

JJ, TKS, MFF, and KS designed the study. LMPH, TKS, MW, EH, and PP performed the experiments. JJ, TKS, DPF, and HW analyzed and interpreted the data. JJ, TKS, DPF, and KS wrote themanuscript, which was corrected, revised and approved by all authors.

### Conflict of interest statement

The authors declare that the research was conducted in the absence of any commercial or financial relationships that could be construed as a potential conflict of interest.
