# Supplementary material for: Production of High Amounts of Hepatotoxin Nodularin and New Protease Inhibitors Pseudospumigins by the Brazilian Benthic Nostoc sp. CENA543
Source: Front Microbiol. 2017 Oct 9;8:1963. doi: 10.3389/fmicb.2017.01963 (PMC5640712; doi:10.3389/fmicb.2017.01963)
Supplement: Supplementary file 1 [file DataSheet1.PDF]

Supporting information

Production of high amounts of hepatotoxin nodularin and new protease inhibitors pseudospumigins by the Brazilian benthic *Nostoc* sp. CENA543

Jouni Jokela,[†] Lassi M. P. Heinilä,[†] Tânia Keiko Shishido,[†] Matti Wahlsten,[†] David P. Fewer,[†] Marli Fátima Fiore,[‡] Hao Wang,[†] Esa Haapaniemi,[§] Perttu Permi,[§,‖] Kaarina Sivonen*,[†]

[†] Department of Food and Environmental Sciences, University of Helsinki, Finland

[‡] Center for Nuclear Energy in Agriculture, University of São Paulo, Piracicaba, Brazil

[§] Department of Chemistry, University of Jyväskylä, Finland

[‖] Department of Biological and Environmental Science, Nanoscience Center, University of Jyväskylä, Finland

Table S1. Ion assignments, calculated (Calc) and experimental (Exp) ion masses and mass differences (Δ) of protonated molecules of pseudospumigins (Psp) A (**3**), C (**5**), D (**6**) and F (**8**) from *Nostoc* sp. CENA543 analyzed by UPLC-QTOF. Spectra are shown in Figure S3.

		Psp A			Psp C			Psp D			Psp F		
		$[M+H]^+$ *(m/z)*		**Δ**	$[M+H]^+$ *(m/z)*		**Δ**	$[M+H]^+$ *(m/z)*		**Δ**	$[M+H]^+$ *(m/z)*		**Δ**
No	Ion assignment	Calc	Exp	(ppm)	Calc	Exp	(ppm)	Calc	Exp	(ppm)	Calc	Exp	(ppm)
1	M + H^+	613,3344	613,3339	-0,9	599,3188	599,3185	-0,5	597,3395	597,3383	-2,1	583,3239	583,3224	-2,6
2	M – H_2O + H^+	595,3239	595,3234	-0,9	581,3082	581,3076	-1,1	579,3289	579,3279	-1,9	565,3133	565,3130	-0,6
3	M – (H_2O + NH_3) + H^+	578,2973	578,2998	4,2	564,2817	564,2835	3,2	562,3024	562,3052	4,9	548,2868	548,2850	-3,3
4	M – 2xH_2O + H^+	577,3133	577,3131	-0,4	563,2976	563,2956	-3,7	561,3184	561,3200	2,8	547,3027	547,2994	-6,2
5	M – (CO+NH_3) + H^+	568,3130	568,3123	-1,2	554,2973	554,3000	4,8	552,3181	552,3170	-2,0	538,3024	538,3089	12,0
6	M – (H_2O+HN=C=NH) + H^+	553,3021	553,3016	-0,9	539,2864	539,2855	-1,8	537,3072	537,3063	-1,7	523,2915	523,2923	1,4
7	M – (2xH_2O+HN=C=NH) + H^+	535,2915	535,2909	-1,2	521,2759	521,2752	-1,3	519,2966	519,2963	-0,7	505,2809	505,2816	1,2
8	Hpla-Hty/Hph-Ile/Val + H^+	455,2177	455,2177	0,0	441,2020	441,2031	2,3	439,2228	439,2223	-1,1	425,2071	425,2077	1,3
9	Hty/Hph-Ile/Val-Argininal + H^+	449,2871	449,2868	-0,7	435,2714	435,2704	-2,5	433,2922	433,2920	-0,5	419,2765	419,2668	-23,3
10	Hty/Hph-Ile/Val-Argininal – H_2O + H^+	431,2765	431,2763	-0,6	417,2609	417,2600	-2,2	415,2816	415,2800	-4,0	401,2660	401,2629	-7,7
11	Hpla-Hty/Hph + H^+	342,1336	342,1338	0,4	342,1336	342,1337	0,1	326,1387	326,1390	0,8	326,1387	326,1377	-3,2
12	Hpla-Hty/Hph – CO + H^+	314,1387	314,1390	0,8	314,1387	314,1386	-0,4	298,1438	298,1435	-1,1	298,1438	298,1435	-1,1
13	Ile/Val-Argininal + H^+	272,2081	272,2085	1,3	258,1925	258,1938	5,0	272,2081	272,2138	20,8	258,1925	258,1973	18,6
14	Hty/Hph-Ile/Val – CO + H^+	263,1754	263,1751	-1,3	249,1598	249,1596	-0,8	247,1805	247,1801	-1,8	233,1648	233,1591	-24,8
15	Ile-Argininal – H_2O + H^+	254,1975	254,1975	-0,4	240,1819	240,1815	-1,8	254,1975	254,1970	-2,3	240,1819	240,1802	-7,2
16	Ile/Val-Argininal – 2xNH_3 + H^+	238,1550	238,1554	1,5	224,1394	224,1402	3,6	238,1550	238,1549	-0,6	224,1394	224,1398	1,8
17	Ile-Argininal – (H_2O+NH_3) + H^+	237,1710	237,1708	-1,0	223,1553	223,1550	-1,7	237,1710	237,1707	-1,4	223,1553	223,1542	-5,3
18	Ile/Val-Argininal – (H_2O+HN=C=NH) + H^+	212,1757	212,1757	-0,4	198,1601	198,1600	-0,7	212,1757	212,1759	0,5	198,1601	198,1611	4,9
19	Hpla-NH_2-CH=CH_2 + H^+	208,0968	208,0969	0,1	208,0968	208,0970	0,6	208,0968	208,1002	16,0	208,0968	208,0940	-13,8
20	Ile-Argininal – (HN=C=NH+H_2O+NH_3) + H^+	195,1492	195,1492	-0,2	181,1335	181,1337	0,6	195,1492	195,1489	-1,7	181,1335	181,1340	2,3
21	Hpla-NH_2 + H^+	182,0812	182,0814	1,0	182,0812	182,0815	1,5	182,0812	182,0813	0,4	182,0812	182,0814	1,0
22	Hpla-NH_2 – H_2O + H^+	164,0706	164,0709	1,5	164,0706	164,0707	0,3	164,0706	164,0707	0,3	164,0706	164,0708	0,9
23	Hty/Hph – CO + H^+	150,0913	150,0915	0,7	150,0913	150,0916	1,4	134,0964	134,0964	-0,6	134,0964	134,0959	-4,3
24	Hpla – H_2O + H^+	147,0441	147,0441	-0,1	147,0441	147,0442	0,6	147,0441	147,0439	-1,4	147,0441	147,0433	-5,5
25	Argininal – NH_3 + H^+	142,0975	142,0979	2,5	142,0975	142,0979	2,5	142,0975	142,0977	1,1	142,0975	142,0986	7,5
26	Argininal – H_2O + H^+	141,1135	141,1139	2,7	141,1135	141,1138	2,0	141,1135	141,1138	2,0	141,1135	141,1130	-3,7
27	Hpla – CO + H^+	137,0597	137,0604	5,0	137,0597	137,0601	2,5	137,0597	137,0599	1,0	137,0597	137,0603	3,9
28	Tyr – CO + H^+	136,0757	136,0759	1,2	136,0757	136,0759	1,2	136,0757	136,0761	2,6	136,0757	136,0759	1,2
29	Hty → HO-C_6H_4-C_3H_3 + H^+;<br>Hph → C_6H_5-C_3H_3 + H^+	133,0648	133,0652	2,7	133,0648	133,0651	1,9	117,0699	117,0700	0,6	117,0699	117,0700	0,6
30	CO-Argininal – (H_2O+HN=C=NH) + H^+	125,0709	125,0711	0,9	125,0709	125,0709	-0,7	125,0709	125,0707	-2,3	125,0709	125,0722	9,7
31	Argininal – (H_2O+NH_3) + H^+	124,0869	124,0870	0,2	124,0869	124,0871	1,0	124,0869	124,0873	2,7	124,0869	124,0861	-7,0
32	HO-C_6H_4-CH=CH_2 + H^+	121,0648	121,0650	1,3	121,0648	121,0651	2,1	121,0648	121,0647	-1,2	121,0648	121,0651	2,1
33	HO-C_6H_4-C≡CH + H^+	119,0491	119,0494	1,8	119,0491	119,0491	-0,8	119,0491	119,0493	0,9	119,0491	119,0487	-4,1
34	HO-C_6H_4-CH_2 + H^+	107,0491	107,0494	1,9	107,0491	107,0494	1,9	107,0491	107,0498	5,7	107,0491	107,0495	2,9
35	Argininal → $C_5H_{10}NO^+$	100,0757	100,0759	1,6	100,0757	100,0759	1,6	100,0757	100,0759	1,6	100,0757	100,0764	6,6
36	Argininal → $C_5H_{11}N_2^+$	99,0917	99,0919	1,8	99,0917	99,0918	0,8	99,0917	99,0918	0,8	99,0917	99,0919	1,8
37	C_6H_4-CH_2 + H^+	91,0542	91,0542	-0,9	91,0542	91,0540	-3,1	91,0542	91,0542	-0,9	91,0542	91,0542	-0,9
38	Ile – CO + H^+	86,0964	86,0964	-0,9	86,0964	86,0965	0,3	86,0964	86,0963	-2,0	86,0964	86,0977	14,2
39	$C_4H_8NO^+$	86,0600	-	-	86,0600	86,0599	-2,2	86,0600	-	-	86,0600	86,0601	0,1
40	Argininal → $C_5H_8N^+$	82,0651	82,0653	1,5	82,0651	82,0651	-1,0	82,0651	82,0651	-1,0	82,0651	82,0648	-4,6
41	Val – CO + H^+	72,0808	-	-	72,0808	72,0810	2,4	72,0808	-	-	72,0808	72,0811	3,7

Table S2. ^{1}H, ^{13}C and ^{15}N NMR data (800 MHz) for pseudospumigin A (**3**) in d_6-DMSO obtained from the analysis of pseudospumigin mixture isolated from *Nostoc* sp. CENA543.

Subunit	C/H No	δC/N (ppm)	δH (ppm)	COSY	HMBC[a]
Hpla[1]	1	173.0			
	2	72.3	4.04	3, 3'	1, 3, 4
	3	39.3	2.65 (2.54)	2, 3'	1, 2, 4, 5/9
	3'		2.87	2, 3	1, 2, 4, 5/9
	4	128.3			
	5	130.6	7.01		2, 3, 6/8, 7
	6	114.6	6.64		4, 6/8, 7
	7	155.6			
Hty[2]	1	171.0			
	2	51.7	4.40	3, 3'	1, 3, 4
	3	34.7	1.79	2, 3', 4, 4'	1, 2, 4, 5
	3'		1.86	2, 3, 4'	1, 2, 4, 5
	4	30.0	2.29	3, 3'	2, 3, 5, 6/10
	4'		2.37	3,3'	2, 3, 5, 6/10
	5	131.3			
	6	128.7	6.89		4, 6/10, 7/9, 8
	7	115.0	6.65		5, 7/9, 8
	8	155.4			
	NH	114.1	7.70 (7.9)		2, Hpla-1
Ile[3]	1	172.7			
	2	56.4	4.14		1, 3, 4, 6
	3	36.5	1.73	6	
	4	24.1	1.09	4', 5	3, 5, 6
	4'		1.40	4, 5	-
	5	10.7	0.79	4, 4'	3,4
	6	15.1	0.82	3	2, 3, 4
	NH	116.6	8.09		2, Hty-1
Argininal[4]	1	75.8	5.29		-
	2	48.8	3.75		-
	3	23.3	1.71		-
	3'		1.71		-
	4	23.3	1.49		-
	4'		1.49		-
	5	38.9	3.12		-
	5'		3.44		-
	6	-			
	2-NH	-			-

- = not detected. Nitrogen shifts referenced to NH_3.

[a] HMBC correlations are from the proton(s) stated to the indicated carbon.

Table S3. BLASTp results of sequences of the enzymes involved in the synthesis of nodularin and pseudospumigin.

Protein	Amino acids	Proposed function	Sequence similarity	Coverage/ Identity (%)	Accession number
**Nodularin biosynthetic enzymes and upstream and downstream open reading frames:**					
ORF 1	556		transglutaminase [*Nostoc* sp. PCC 7524]	100/91	WP_015136911.1
			alr3376 [*Nostoc* sp. PCC 7120]	100/86	BAB75075.1
ORF 2	956		magnesium-transporting ATPase [*Nostoc* sp. PCC 7524]	100/88	WP_015136912.1
			cation-transporting ATPase [*Nostoc* sp. NIES-3756]	100/86	BAT55636.1
ORF 3	375		DNA replication/repair protein RecF [*Nostoc* sp. PCC 7524]	98/85	WP_015136913.1
			DNA replication/repair protein RecF [*Nostoc* sp. PCC 7107]	98/84	WP_015111253.1
ORF 4	142		conserved hypothetical protein [*Anabaena variabilis* ATCC 29413]	96/66	ABA22997.1
			hypothetical protein [*Nostoc* sp. PCC 7120]	96/66	WP_010997524.1
NdaA	2627	NRPS	NdaA [*Nostoc* sp. 73.1]	99/83	AEO14743.1
			non-ribosomal peptide synthetase [*Nodularia spumigena* CCY9414]	99/78	WP_017804328.1
NdaB	1284	NRPS	non-ribosomal peptide synthetase [*Anabaena* sp. 90]	99/88	WP_015078941.1
			NdaB [*Nostoc* sp. 73.1]	99/83	AEO14744.1

NdaC	2652	NRPS-PKS	peptide synthetase polyketide synthase fusion protein McyG [*Anabaena* sp. 90]	99/88	WP_015078938.1
			NdaC [*Nodularia spumigena* NSOR10]	99/79	AAO64404.1
NdaD	3903	PKS	polyketide synthase [*Anabaena* sp. 90]	100/89	WP_015078937.1
			type I polyketide synthase [*Fischerella* sp. PCC 9339]	99/79	WP_035114367.1
NdaE	309	O-methyltransferase	putative O-methyl transferase [*Anabaena* sp. 90]	100/97	AAO62583.1
			methyltransferase [*Anabaena* sp. 90]	100/96	WP_041458256.1
NdaF	3483	NRPS/PKS	hybrid non-ribosomal peptide synthetase/type I polyketide synthase [*Anabaena* sp. 90]	100/94	WP_015078935.1
			polyketide synthase peptide sythetase fusion protein [*Anabaena* sp. 90]	100/94	AAO62582.1
NdaG	265	racemase	Asp/Glu racemase McyF [*Anabaena* sp. 90]	95/94	WP_015078934.1
			McyF [*Nostoc* sp. 152]	99/76	AGZ05271.1
NdaH	336	dehydrogenase	dehydrogenase McyI [*Anabaena* sp. 90]	100/93	WP_015078933.1
			dehydrogenase [*Mastigocladus laminosus* UU774]	99/82	WP_044451333.1
NdaI	606	ABC transporter	ABC transporter ATP-binding protein [*Anabaena* sp. 90]	97/94	WP_015078932.1

			ABC transporter ATP-binding-like protein [*Anabaena* sp. 90]	97/94	AAO62579.1
ORF 5	565		hypothetical protein [*Nostoc* sp. PCC 7524]	97/82	WP_015137213.1
			glycosyl transferase [*Cylindrospermum stagnale* PCC7417]	97/74	WP_051056130.1
ORF 6	414	sulfonate ABC transporter permease	dolichol-phosphate mannosyltransferase [*Nostoc* sp. NIES-3756]	100/80	BAT51165.1
			hypothetical protein [*Nostoc* sp. PCC 7524]	100/86	WP_015137216.1
ORF 7	387	FO synthase	FO synthase subunit 2 [*Nostoc* sp. PCC 7107]	96/86	AFY44209.1
			FO synthase subunit 2 [*Nostoc* sp. PCC 7524]	97/85	AFY46761.1
**Pseudospumigins biosynthetic enzymes and upstream and downstream open reading frames:**					
ORF 1	693		S-layer protein [*Nostoc* sp. PCC 7524]	100/81	WP_015138534.1
			hypothetical protein [*Nostoc* sp. PCC 7120]	100/81	WP_010995786.1
ORF 2	111		XisI protein [*Fischerella muscicola* SAG 1427-1 = PCC 73103]	100/76	WP_016860372.1
			XisI protein [*Nodularia spumigena* CCY9414]	100/74	WP_006196511.1
ORF 3	732		signal transduction protein [*Nostoc* sp. NIES-3756]	98/75	BAT53248.1
			histidine kinase [*Anabaena* sp. MDT14b]	98/66	OBQ38198.1
SpuA	1396	NRPS	AMP-dependent synthetase [*Nostoc piscinale* CENA21]	100/79	WP_062292587.1

			AMP-dependent synthetase [*Nostoc piscinale* CENA21]	97/79	ALF56360.1
SpuB	4116	NRPS	Non-ribosomal peptide synthetase [*Nodularia spumigena* CCY9414]	100/80	WP_006198652.1
			Non-ribosomal peptide synthetase [*Nodularia spumigena* CENA596]	100/80	WP_063874476.1
SpuF	667	ABC transporter	ABC transporter [*Aphanizomenon flos-aquae* NIES-81]	100/78	WP_027403938.1
			ABC transporter [*Nostoc* sp. NIES-3756]	100/74	BAT54532.1
ORF 4	558		poly(A) polymerase [*Tolypothrix* sp. PCC 7601]	99/83	WP_045869381.1
			poly(A) polymerase [*Tolypothrix* sp. PCC 7601]	99/75	WP_045871542.1
ORF 5	200		transposase [*Tolypothrix* sp. PCC 7601]	97/80	WP_045869452.1
			transposase [*Chlorogloeopsis fritschii* PCC 6912]	97/75	WP_016879483.1

Table S4. Binding pockets of the adenylation domains present in the biosynthetic gene clusters of nodularin and pseudospumigin in *Nostoc* sp. CENA543.

Compound	Protein	Adenylation domain binding pocket											Prediction of substrate	Activated substrate
		235	236	239	278	299	301	322	330	331	517	score (%)		
Nodularin	NdaA_A1	D	F	W	N	I	G	M	V	H	K	100	Thr	Thr
	NdaA_A2	D	A	R	H	V	G	I	F	V	K	60	Tyr	MeAsp
	NdaB	D	A	W	S	F	G	L	V	D	K	80	Gln	Arg
	NdaC*	V	G	V	W	V	A	G	S	K	K	-[¤]	-[¤]	Phenyl-acetate
	NdaD*	-	-	-	-	-	-	-	-	-	-	-	-	Malonyl-CoA
	NdaF*	D	P	R	H	S	G	V	V	G	K	100	Glu	Glu
Pseudospumigin	SpuA	V	G	V	W	I	A	A	S	G	K	-[¤]	-[¤]	Hpla
	SpuB_A1	D	L	A	F	T	G	C	V	T	K	60	Leu	Hty
	SpuB_A2	D	A	F	F	L	G	V	T	F	K	100	Ile	Ile/Leu/Val
	SpuB_A3	D	V	E	T	T	G	A	V	T	K	70	Arg	Arg

* PKS AT prediction: malonyl-CoA

[¤] Manual alignment and no prediction was added

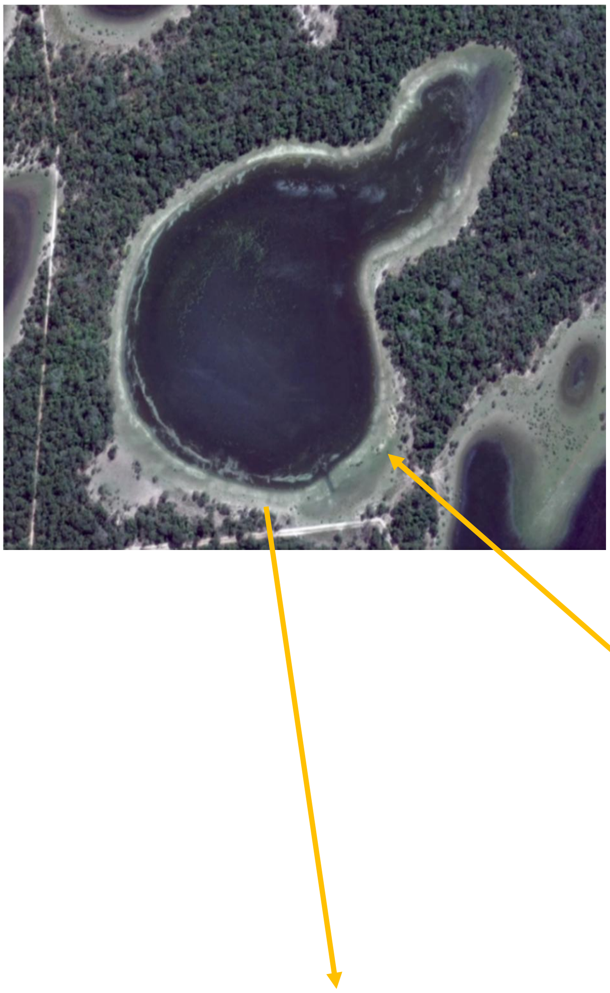

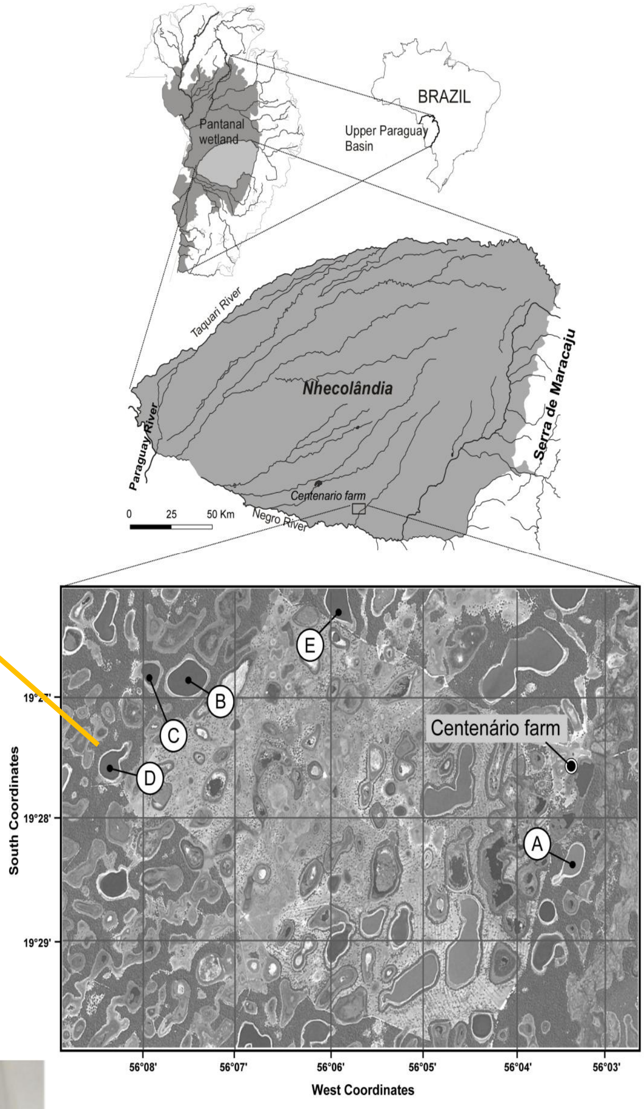

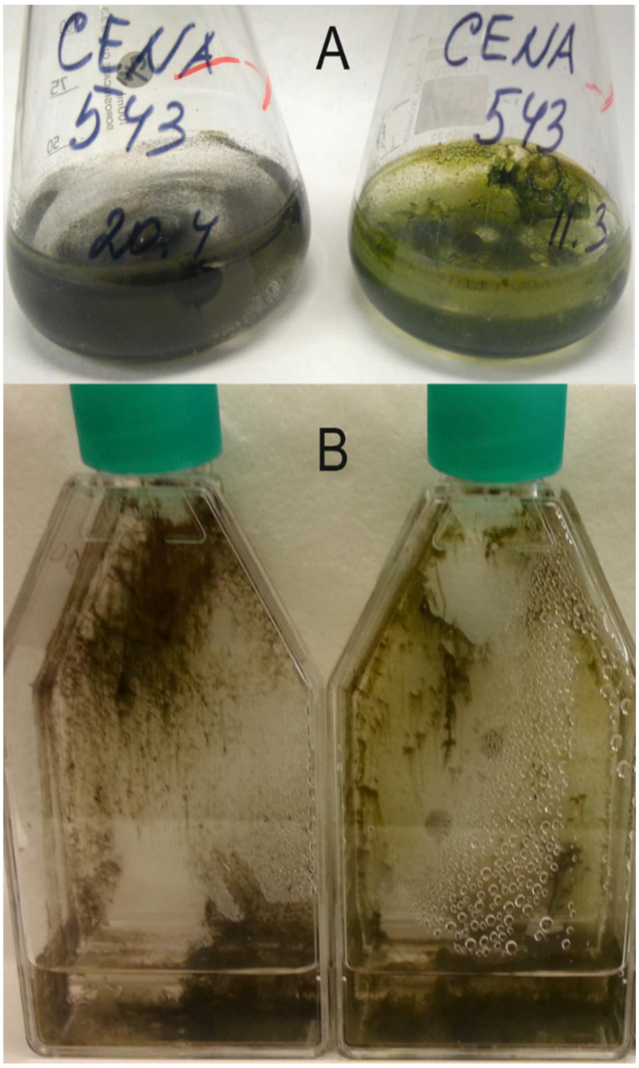

Figure S1. Location of the studied sites in the Nhecolândia, Pantanal wetland area of Brazil, and growth of the *Nostoc* sp. CENA543 on 0.6‰ salinity Z8 medium with no shaking (A) or with mild horizontal shaking (B). In panel A the left culture is one month old and the right culture is over two months old. (A) Salina Verde, (B) Salina Grande, (C) Salina Preta, (D) Salina 67 mil and (E) Salina Centenário.

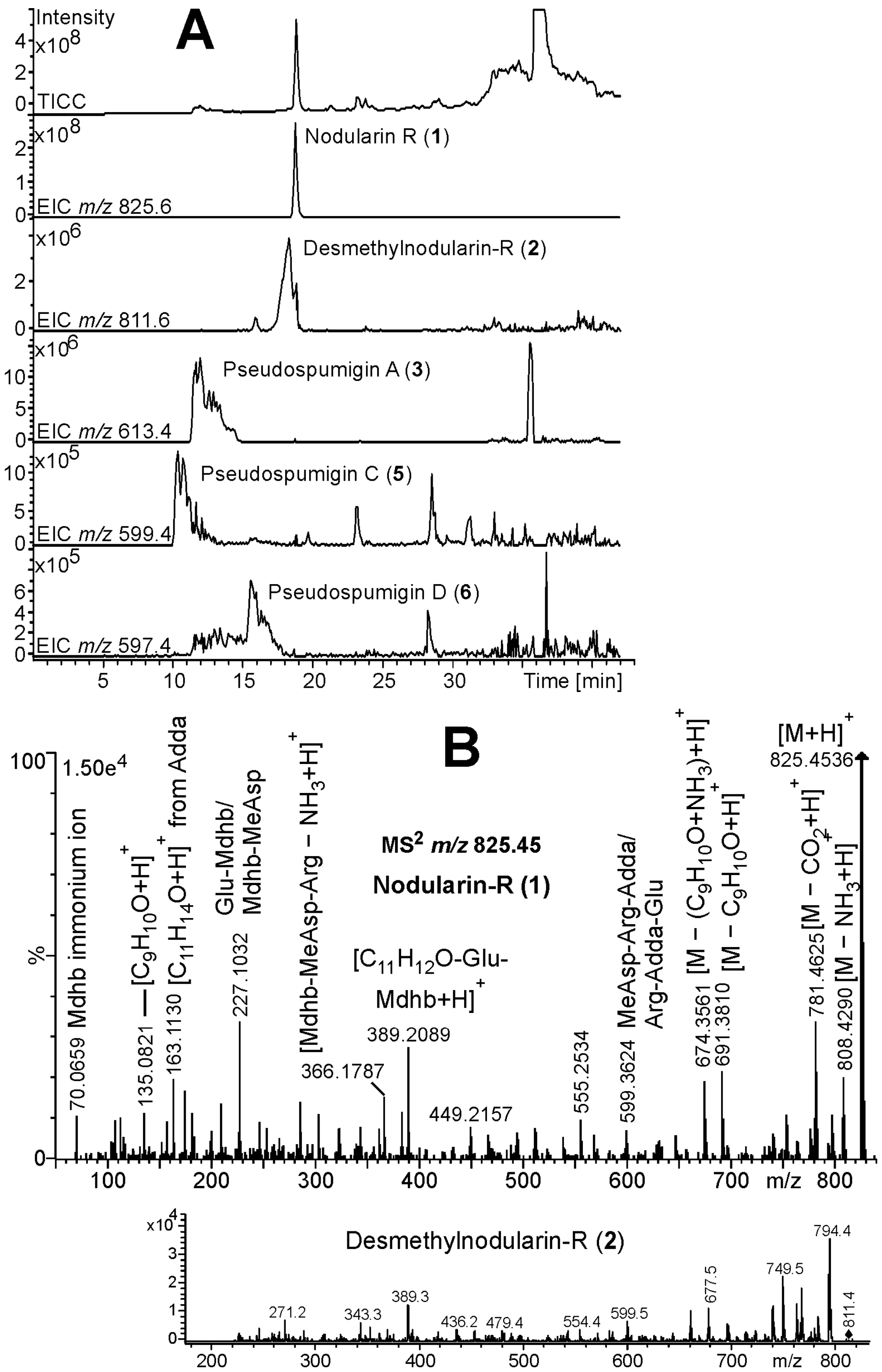

Figure S2. LC-ITMS total ion current (TICC) and ion chromatograms (EIC) of NODs (*m/z* 825.6 and *m/z* 811.6) and Psps (*m/z* 613.4, *m/z* 599.4 and *m/z* 597.4) in panel A and partially annotated accurate mass product ion spectra of protonated nodularin-R (**1**) (*m/z* 825.45, $[M+H]^+$) and product ion spectra of protonated desmethylnodularin-R (**2**) (*m/z* 811.43, $[M+H]^+$) in panel B from *Nostoc* sp. CENA543.

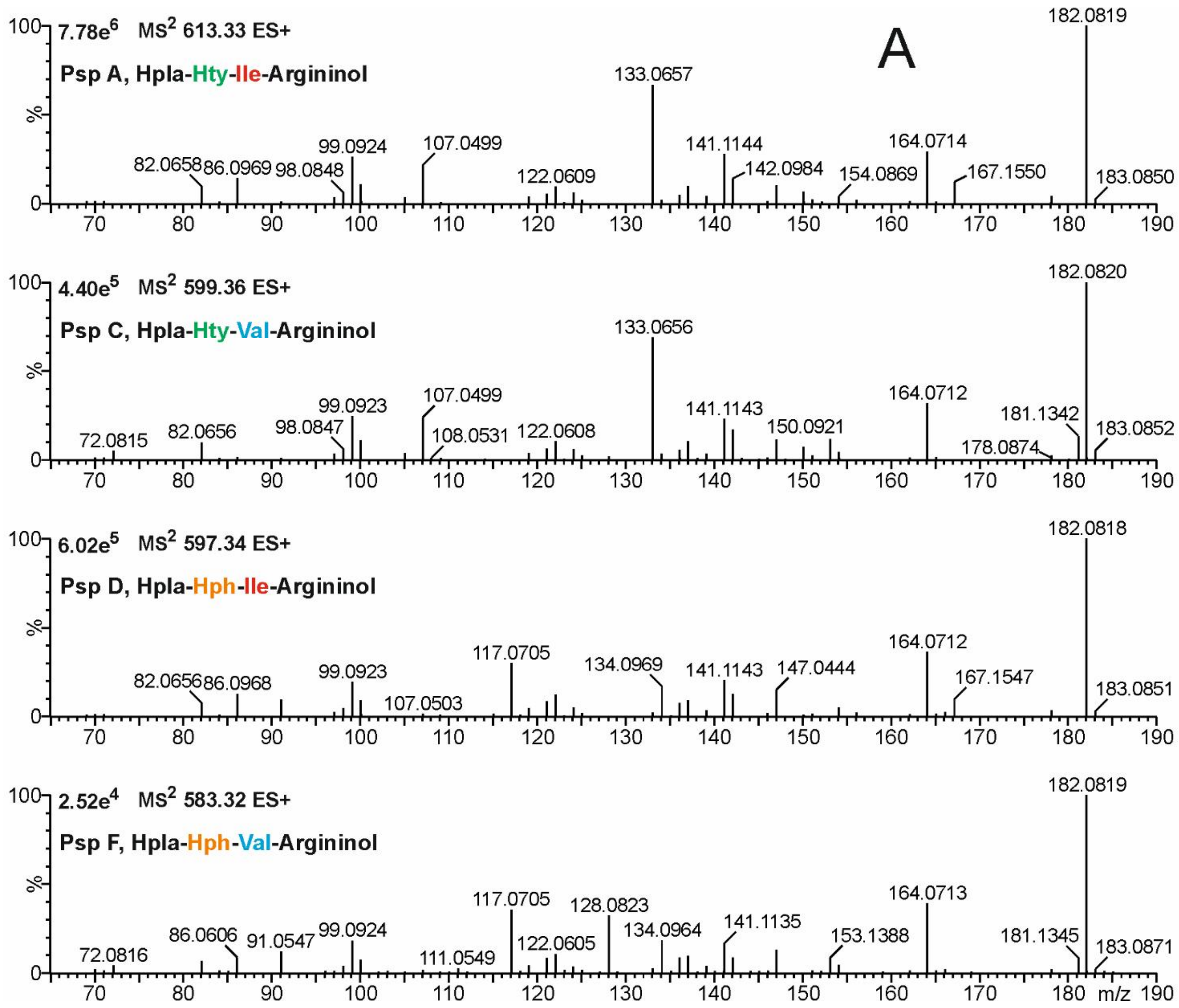

$[M+H]^+$	Ion assignment
182.08	Hpla-NH_2 + H^+
181.13	Val-Argininal – (HN=C=NH+H_2O+NH_3) + H^+
164.07	Hpla-NH_2 – H_2O + H^+
150.09	Hty – CO + H^+
147.04	Hpla – H_2O + H^+
142.10	Argininal – NH_3 + H^+
141.11	Arginina – H_2O + H^+
137.06	Hpla – CO + H^+
136.08	Tyr – CO + H^+
134.10	Hph – CO + H^+
133.06	Hty → HO-C_6H_4-C_3H_3 + H^+
125.07	CO-Argininal – (H_2O+HN=C=NH) + H^+
124.09	Argininal – (H_2O+NH_3) + H^+
121.06	HO-C_6H_4-CH=CH_2 + H^+
119.05	HO-C_6H_4-C≡CH + H^+
117.07	Hph → C_6H_5-C_3H_3 + H^+
107.05	HO-C_6H_4-CH_2 + H^+
100.08	Argininal → $C_5H_{10}NO^+$
99.09	Argininal → $C_5H_{11}N_2^+$
91.05	C_6H_4-CH_2 + H^+
86.10	Ile – CO + H^+
86.06	$C_4H_8NO^+$
82.07	Argininal → $C_5H_8N^+$
72.08	Val – CO + H^+

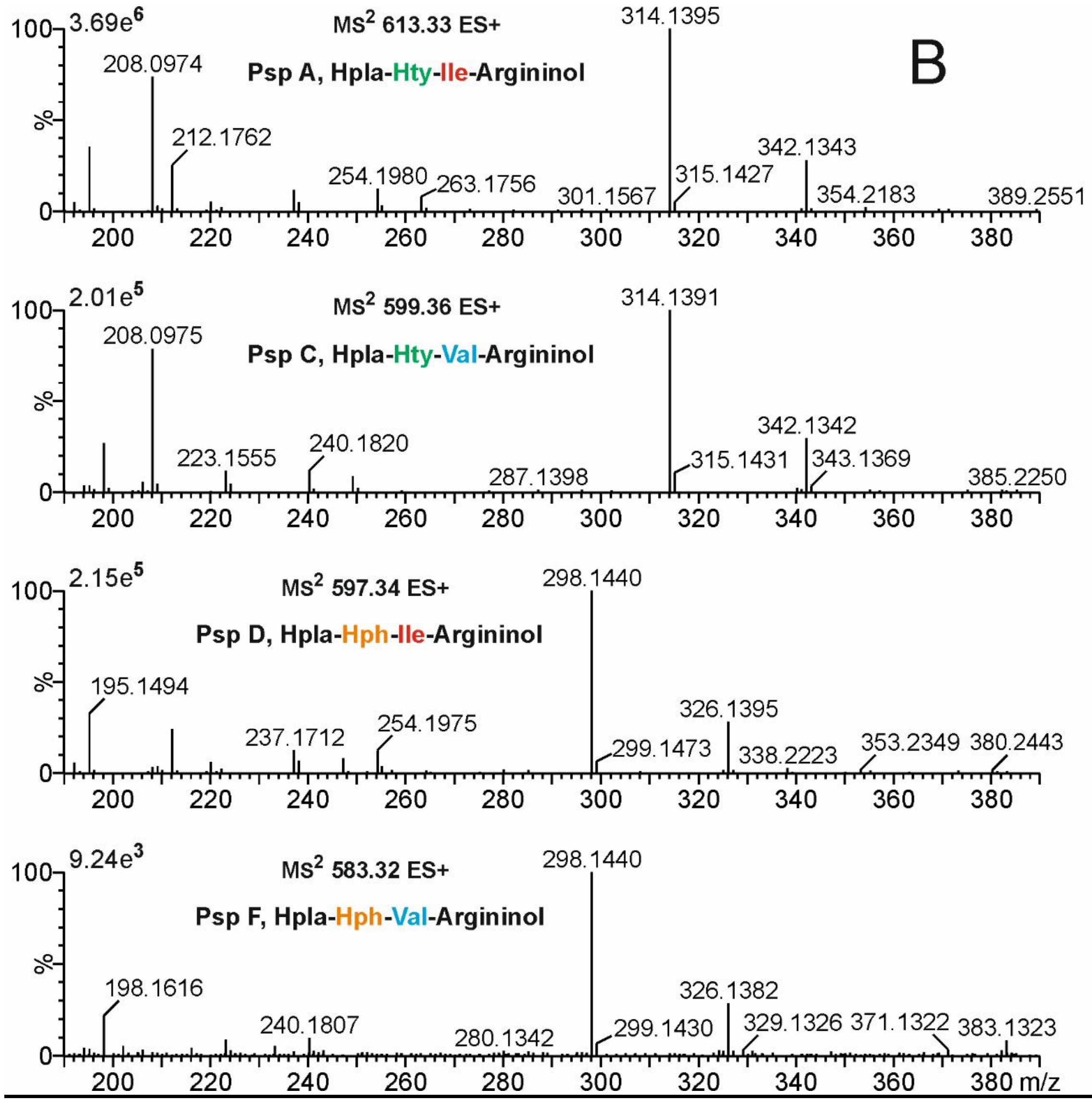

$[M+H]^+$	Ion assignment
342.13	Hpla-Hty + H^+
326.14	Hpla-Hph + H^+
314.14	Hpla-Hty − CO + H^+
298.14	Hpla-Hph − CO + H^+
272.21	Ile-Argininal + H^+
263.18	Hty-Ile − CO + H^+
258.19	Val-Argininal + H^+
254.20	Ile-Argininal − H_2O + H^+
249.16	Hty-Val − CO + H^+
247.18	Hph-Ile − CO + H^+
240.18	Val-Argininal − H_2O + H^+
238.16	Ile-Argininal − $2xNH_3$ + H^+
237.17	Ile-Argininal − (H_2O+NH_3) + H^+
233.16	Hph-Val − CO + H^+
224.14	Val-Arginina − $2xNH_3$ + H^+
223.16	Val-Argininal − (H_2O+NH_3) + H^+
212.18	Ile-Argininal − (H_2O+HN=C=NH) + H^+
208.10	Hpla-NH_2-CH=CH_2 + H^+
198.16	Val-Argininal − (H_2O+HN=C=NH) + H^+
195.15	Ile-Argininal − (HN=C=NH+H_2O+NH_3) + H^+

[M+H]$^+$ (m/z)					[M+H]$^+$ (*m/z*)				
Psp A	Psp C	Psp D	Psp F	Ion assignment	Psp A	Psp C	Psp D	Psp F	Ion assignment
553.30	539.29	537.31	523.29	M − (H_2O+HN=C=NH) + H^+	613.33	599.32	597.34	583.32	M + H^+
535.29	521.28	519.30	505.28	M − (2xH_2O+HN=C=NH) + H^+	595.32	581.31	579.33	565.31	M − H_2O + H^+
455.22	441.20	439.22	425.21	Hpla-Hty/Hph-Ile/Val + H^+	578.30	564.28	562.30	548.29	M − (H_2O + NH_3) + H^+
449.29	435.27	433.29	419.28	Hty/Hph-Ile/Val-Argininal + H^+	577.31	563.30	561.32	547.30	M − 2xH_2O + H^+
431.28	417.26	415.28	401.27	Hty/Hph-Ile/Val-Argininal − H_2O + H^+	568.31	554.30	552.32	538.30	M − (CO+NH_3) + H^+

C

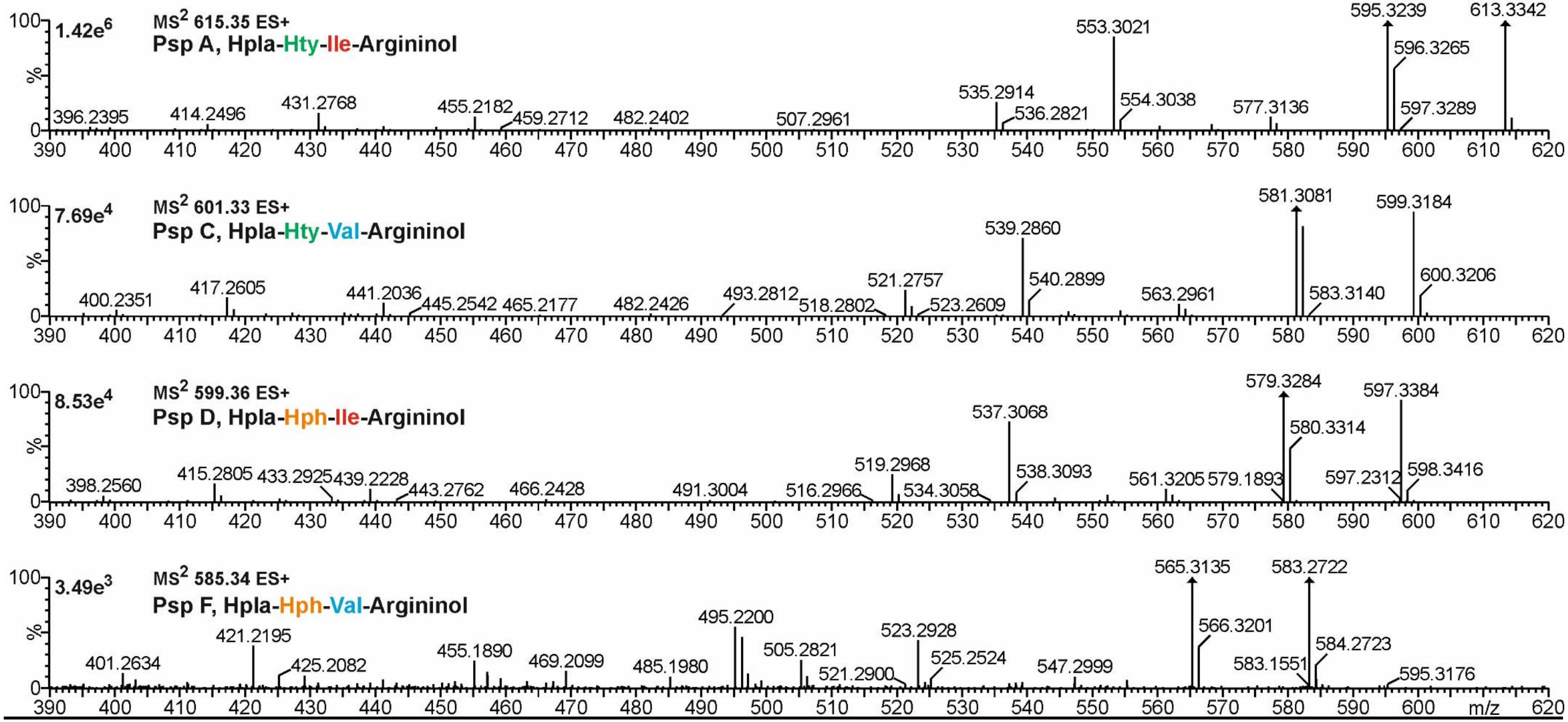

$[M+H]^+$	Ion assignment	$[M+H]^+$	Ion assignment	$[M+H]^+$	Ion assignment	$[M+H]^+$	Ion assignment	$[M+H]^+$	Ion assignment
72.08	Val − CO + H^+	101.11	Argininol → $C_5H_{13}N_2^+$	119.05	HO-C_6H_4-C≡CH + H^+	134.10	Hph − CO + H^+	147.04	Hpla − H_2O + H^+
84.08	Argininol → $C_5H_{10}N^+$	102.09	Argininol → $C_5H_{12}NO^+$	121.06	HO-C_6H_4-CH=CH_2 + H^+	136.08	$C_8H_{10}NO^+$	150.09	Hty − CO + H^+
86.10	Ile − CO + H^+	107.05	HO-C_6H_4-CH_2 + H^+	127.09	CO-Argininol − (H_2O+HN=C=NH) + H^+	137.06	Hpla − CO + H^+	155.08	Hpla-Hty/Hph-Ile/Val → $C_7H_{11}N_2O_2^+$
91.05	C_6H_4-CH_2 + H^+	117.07	Hph → C_6H_5-C_3H_3 + H^+	133.06	Hty → HO-C_6H_4-C_3H_3 + H^+	144.11	Argininol − NH_3 + H^+	164.07	Hpla-NH_2 − H_2O + H^+
								182.08	Hpla-NH_2 + H^+

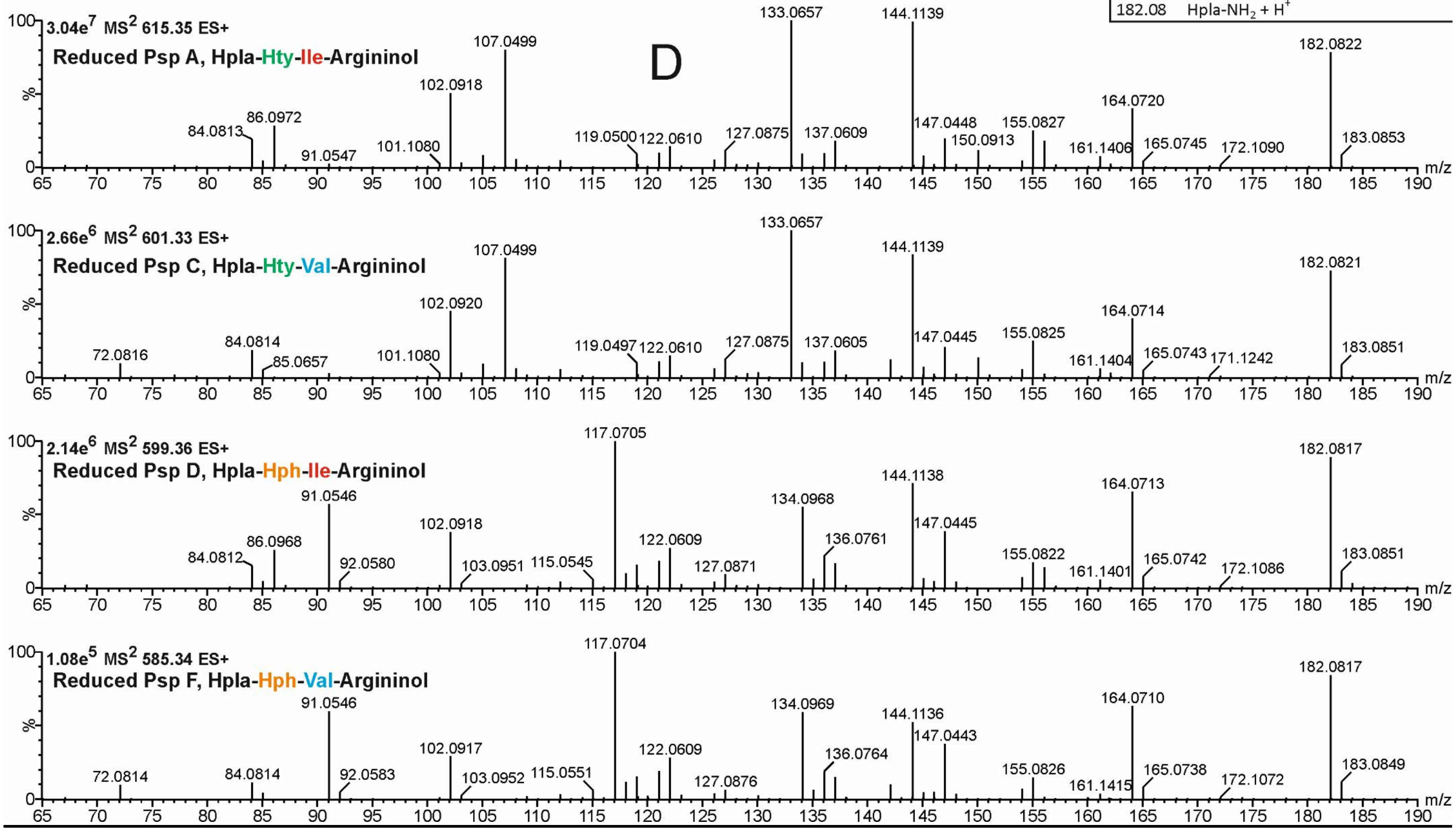

$[M+H]^+$	Ion assignment
200.18	Val-Argininol – (H_2O+HN=C=NH) + H^+
208.10	Hpla-NH_2-CH=CH_2 + H^+
214.19	Ile-Argininol – (H_2O+HN=C=NH) + H^+
218.19	Val-Argininol – HN=C=NH + H^+

$[M+H]^+$	Ion assignment
226.16	Val-Argininol – 2xNH_3 + H^+
232.20	Ile-Argininol – HN=C=NH + H^+
233.16	Hph-Val – CO + H^+
240.17	Ile-Argininol – 2xNH_3 + H^+

$[M+H]^+$	Ion assignment
243.18	Val-Argininol – NH_3 + H^+
247.18	Hph-Ile – CO + H^+
249.16	Hty-Val – CO + H^+
257.20	Ile-Argininol – NH_3 + H^+

$[M+H]^+$	Ion assignment
260.21	Val-Argininol + H^+
263.18	Hty-Ile – CO + H^+
274.22	Ile-Argininol + H^+
298.14	Hpla-Hph – CO + H^+

$[M+H]^+$	Ion assignment
314.14	Hpla-Hty – CO + H^+
326.14	Hpla-Hph + H^+
342.13	Hpla-Hty + H^+

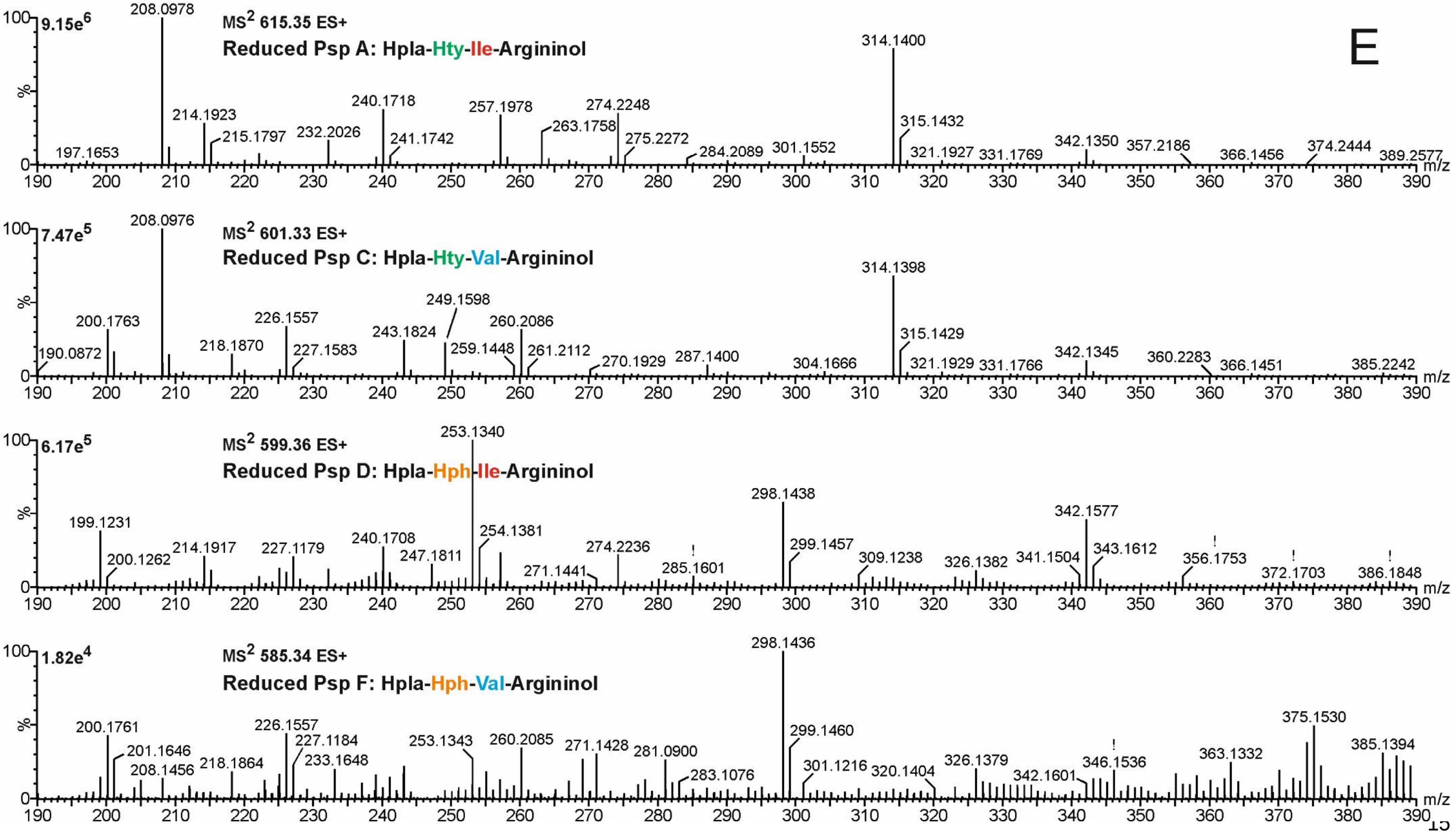

Ion assignment reduced:	[M+H]$^+$ Psp A	Psp C	Psp D	Psp F
Hty/Hph-Ile/Val-Argininol − H_2O + H^+	433.29	419.28	417.30	403.28
Hty/Hph-Ile/Val-Argininol − NH_3 + H^+	434.28	420.26	418.28	404.27
Hty/Hph-Ile/Val-Argininol + H^+	451.30	437.29	435.31	421.29
M − (HN=C(NH_3)$_2$ + H_2O) + H^+	538.29	524.28	522.30	508.28

Ion assignment	[M+H]$^+$ Psp A	Psp C	Psp D	Psp F
M − (H_2O+HN=C=NH) + H^+	555.32	541.30	539.32	525.31
M − HN=C(NH_3)$_2$ + H^+	556.30	542.29	540.31	526.29
M − HN=C=NH + H^+	573.33	559.31	557.33	543.32
M − (H_2O + NH_3) + H^+	580.31	566.30	564.32	550.30

Ion assignment	[M+H]$^+$ Psp A	Psp C	Psp D	Psp F
M − 2xNH_3 + H^+	581.30	567.28	565.30	551.29
M − H_2O + H^+	597.34	583.32	581.34	567.33
M − NH_3 + H^+	598.32	584.31	582.33	568.31
M + H^+	615.35	601.33	599.36	585.34

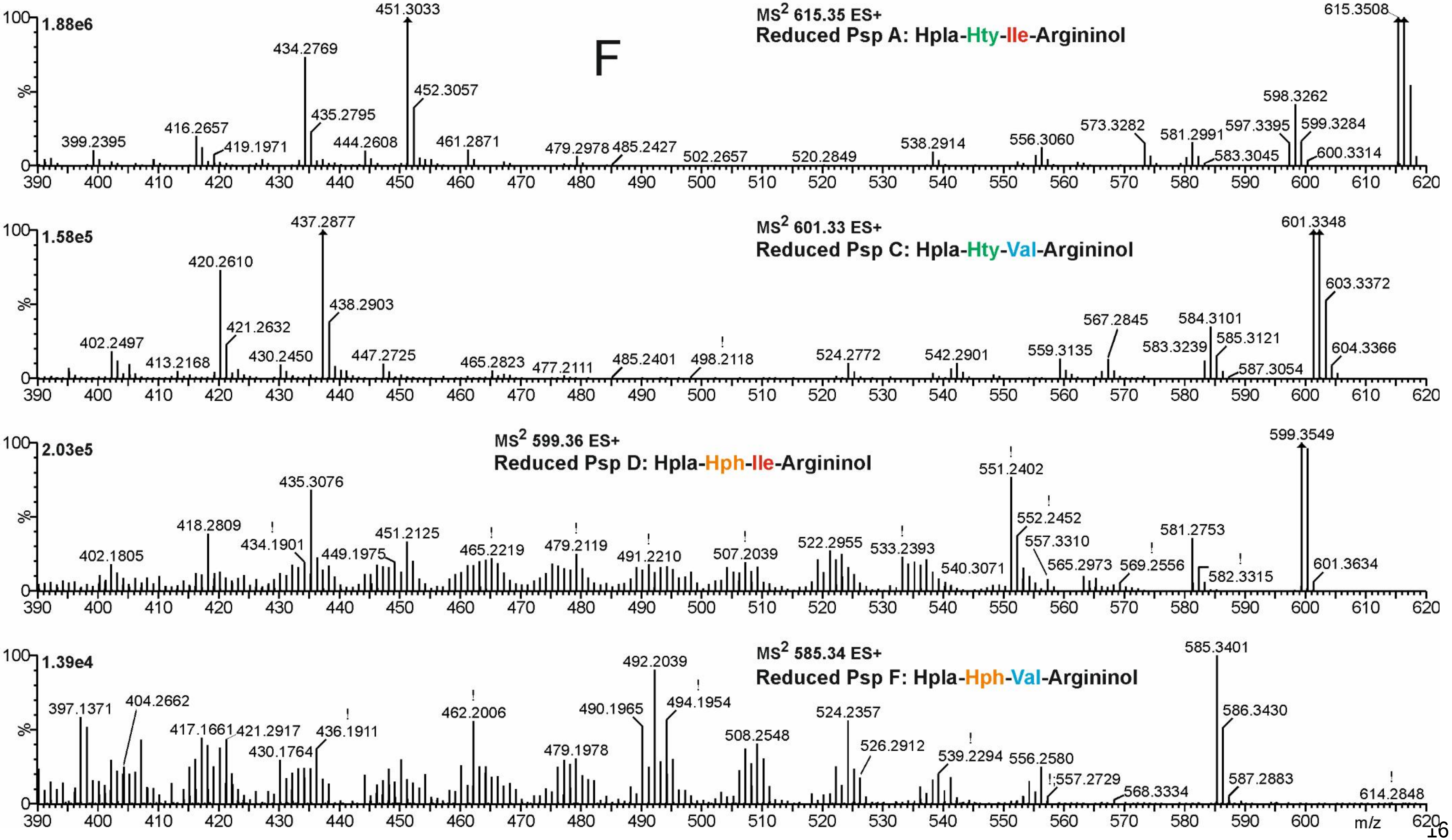

Figure S3. High resolution QTOF product ion spectra of protonated pseudospumigins (Psp) A (**3**), C (**5**), D (**6**) and F (**8**) in panels A, B and C and corresponding $NaBH_4$ reduced pseudospumigins in panels D, E and F. Ions *m/z* 65–190 in panel A and D, ions *m/z* 190–390 in panel B and E and ions *m/z* 390–620 in panel C and F. Ion assignments are presented in Table S1.

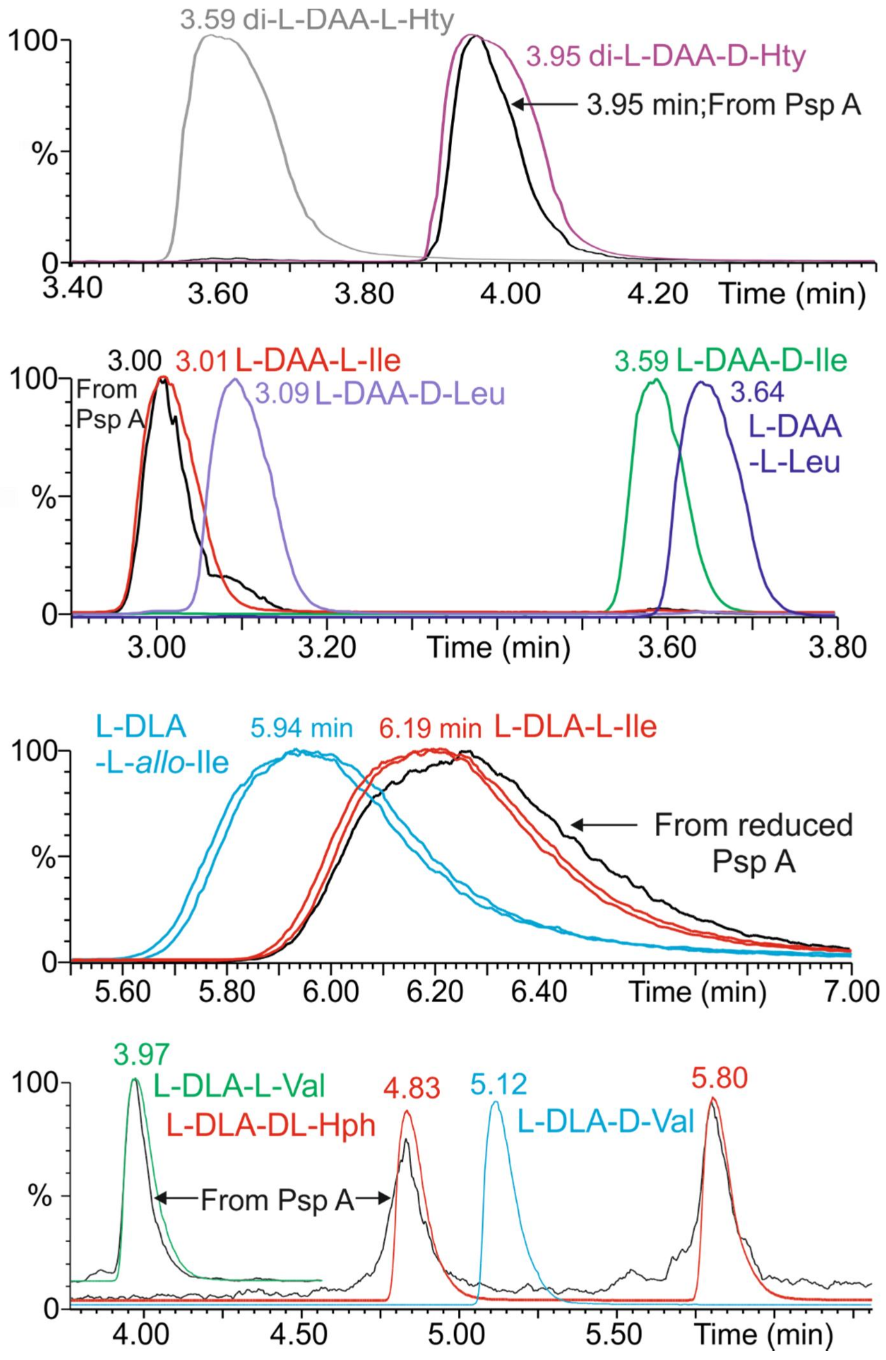

Figure S4. Amino acid analysis of first $NaBH_4$ reduced and then acid hydrolyzed pseudospumigin A mixture (Psp A). UPLC-QTOF ion chromatogram peaks of L-DAA (2,4-dinitrophenyl-5-L-alanine amide) and L-DLA (2,4-dinitrophenyl-5-L-leucine amide) derivatives of L-Hty, D-Hty, L-Ile, D-Ile, L-allo-Ile, L-Leu, D-Leu, L-Val, D-Val and L/D-Hph standards (colored traces) and amino acids from Psp A acid hydrolysate (black traces).

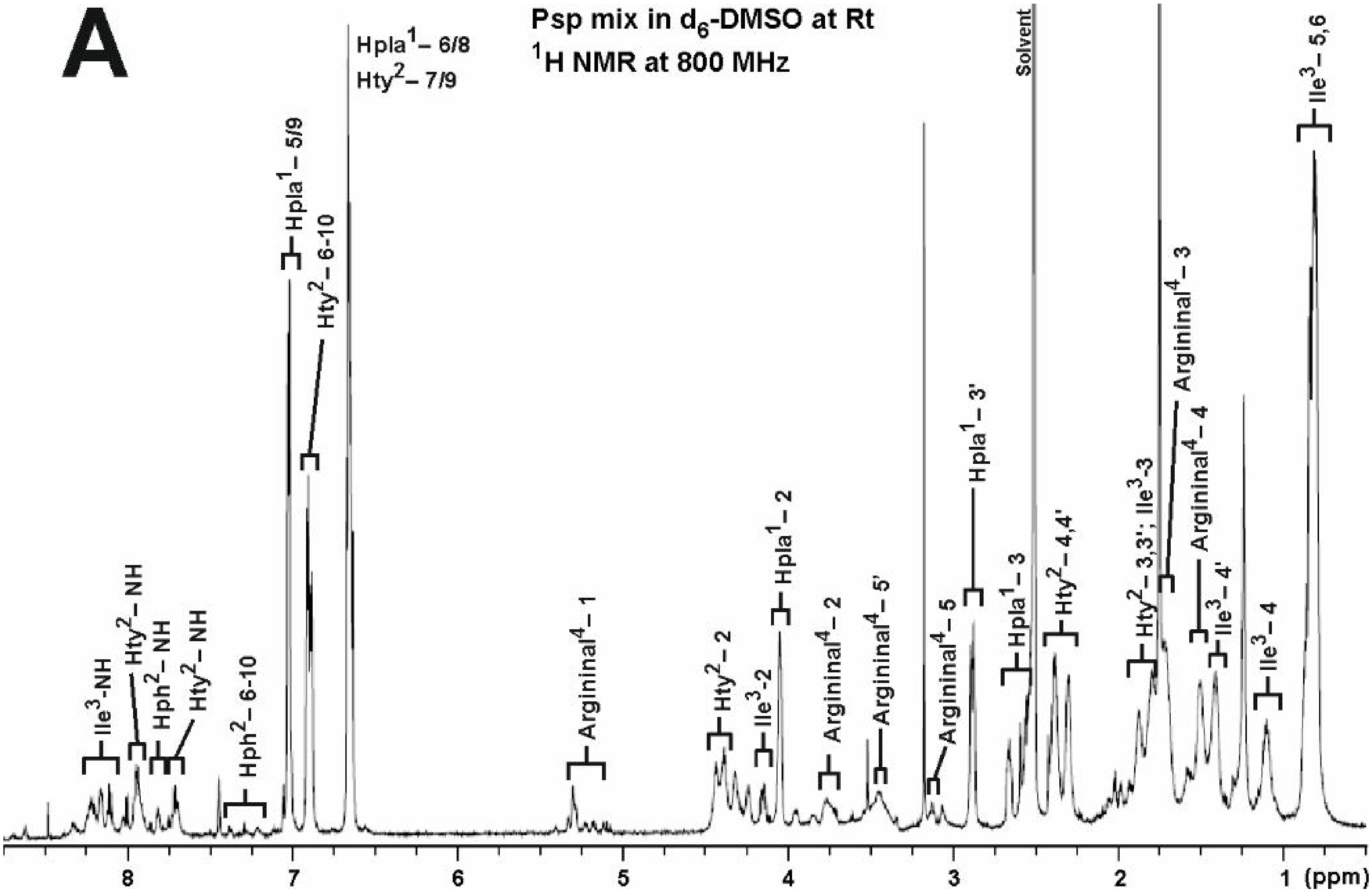
A
Psp mix in d6-DMSO at Rt
1H NMR at 800 MHz
Hpla1– 6/8
Hty2– 7/9
Solvent
Ile3– 5,6
Hpla1– 5/9
Hty2– 6-10
Argininal4– 3
Argininal4– 4
Hpla1– 3'
Hty2– 3,3'; Ile3-3
Hty2– 4,4'
Ile3– 4'
Hpla1– 2
Hpla1– 3
Argininal4– 5
Argininal4– 5'
Argininal4– 2
Ile3– 4
Hty2– NH
Hph2– NH
Hty2– NH
Ile3-NH
Hph2– 6-10
Argininal4– 1
Hty2– 2
Ile3-2
8
7
6
5
4
3
2
1 (ppm)

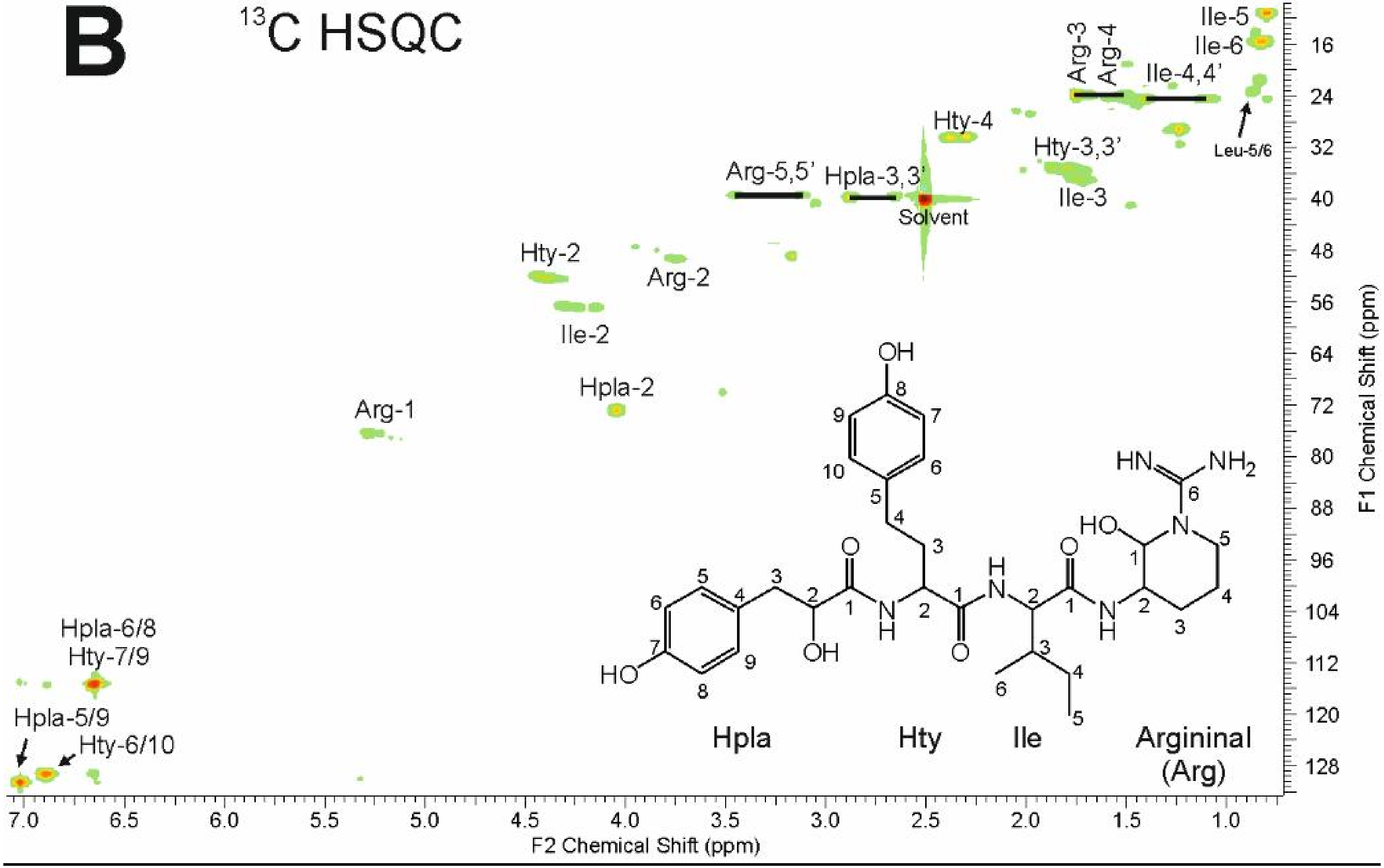
B
13C HSQC
Ile-5
Ile-6
Arg-3
Arg-4
Ile-4,4'
Leu-5/6
Hty-4
Hty-3,3'
Arg-5,5'
Hpla-3,3'
Solvent
Ile-3
Hty-2
Arg-2
Ile-2
Hpla-2
Arg-1
Hpla-6/8
Hty-7/9
Hpla-5/9
Hty-6/10
Hpla
Hty
Ile
Argininal
(Arg)
F2 Chemical Shift (ppm)
F1 Chemical Shift (ppm)

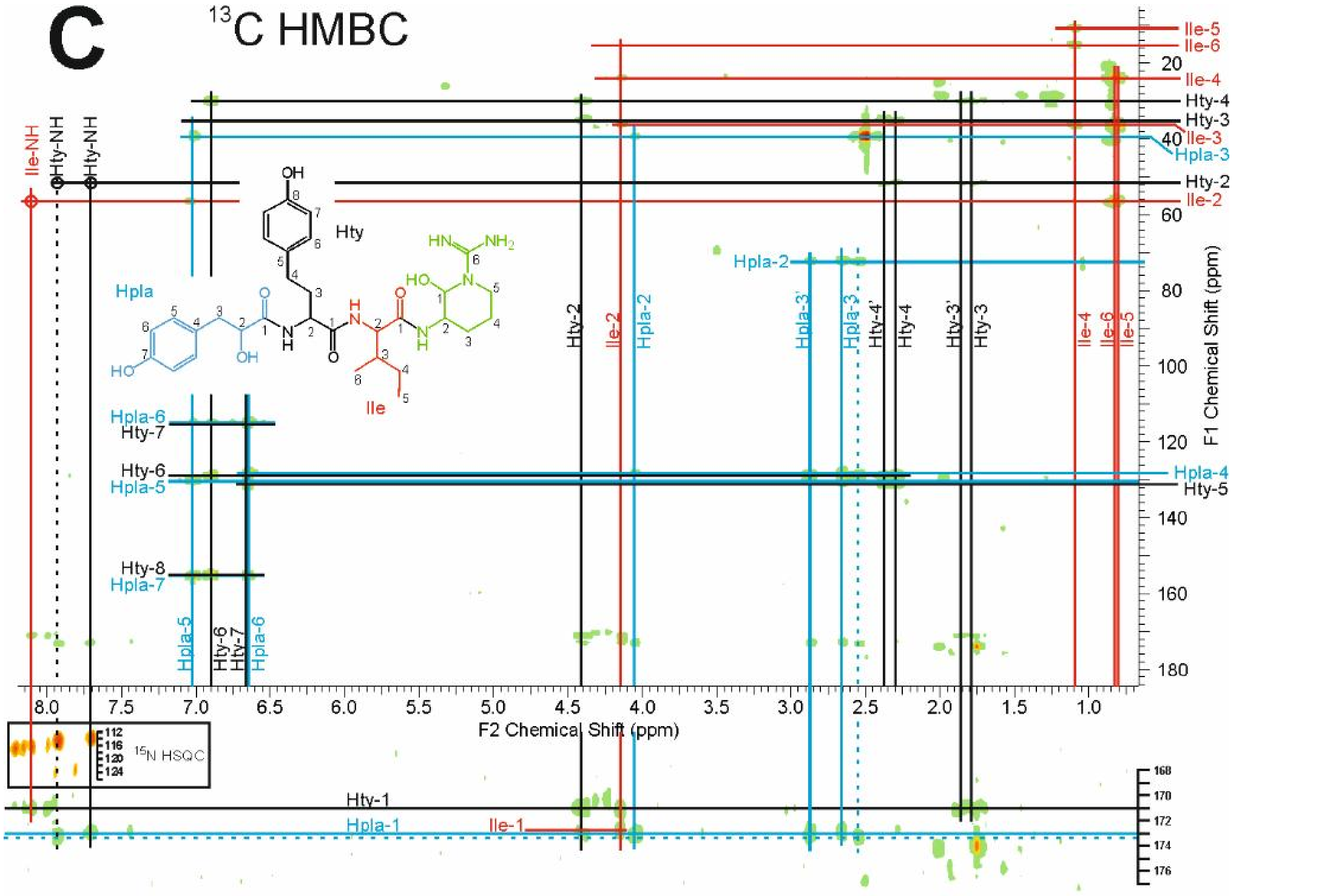

Figure S5. 800 MHz NMR spectra of the pseudospumigin (Psp) mixture that were isolated from *Nostoc* sp. CENA543. A: ^{1}H, B: ^{1}H-^{13}C HSQC, C: ^{1}H-^{13}C HMBC and ^{1}H-^{15}N HSQC. O = signal weak.

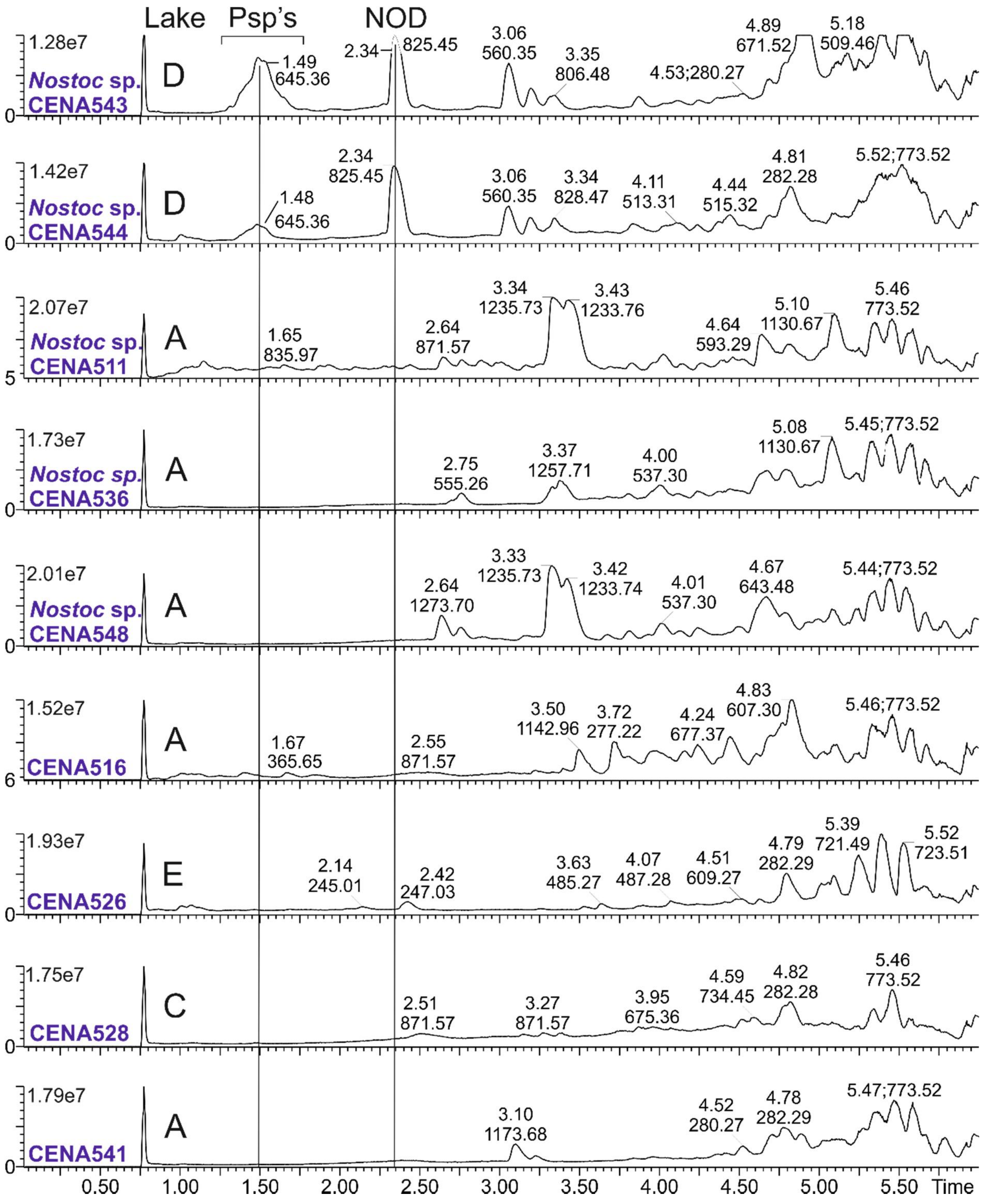

<u>Figure S6.</u> UPLC-QTOF total ion current chromatograms of methanol extracts of cyanobacteria collected from Brazilian lakes (see Figure S1). Non *Nostoc* strains are *Pantanalinema rosaneae* CENA 516, *Geminocystis* sp. CENA526, *Alkalinema pantanalense* CENA528 and *Tolypothrichaceae* CENA541. *A. pantanalense* CENA531 was very similar with *A. pantanalense* CENA528.

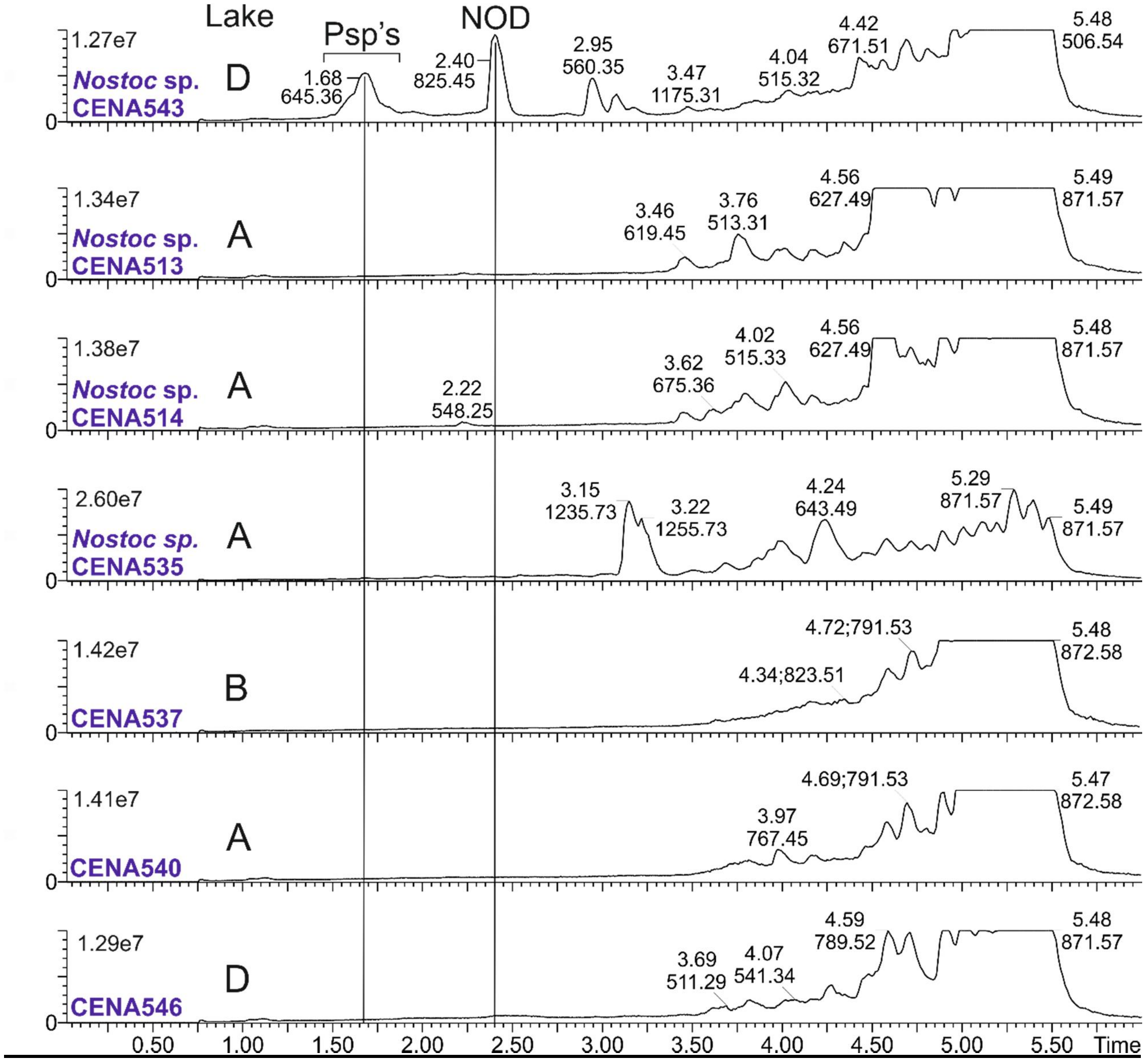

Figure S7. UPLC-QTOF total ion current chromatograms of methanol extracts of cyanobacteria collected from Brazilian lakes (see Figure S1). Non *Nostoc* strains are *Pantanalinema rosaneae* CENA 537, *Leptolyngbya* sp. CENA540 and *Nodosilinea* sp. CENA546. *P. rosaneae* CENA 517 was very similar with *P. rosaneae* CENA 537.
